# Heterologous mucosal vaccine boosting enhances mucosal and systemic immunity by distinct mechanisms

**DOI:** 10.1084/jem.20241529

**Published:** 2025-10-30

**Authors:** Cameron Bissett, Lyn Yong, Alexandra J. Spencer, Fionn Nok Lam Ma, Ethan A. Courchesne, Reshma Koolaparambil Mukesh, Marta Ulaszewska, Alexander Sampson, Marie Lucienne, Reshma Kailath, Susan Morris, Claire Powers, Sandra Belij-Rammerstorfer, Vincent J. Munster, Neeltje van Doremalen, Nicholas M. Provine, Teresa Lambe

**Affiliations:** 1 Department of Paediatrics, Oxford Vaccine Group, University of Oxford, Oxford, UK; 2 Pandemic Sciences Institute, University of Oxford, Oxford, UK; 3 Centre for Human Genetics, University of Oxford, Oxford, UK; 4 School of Biomedical Sciences and Pharmacy, University of Newcastle, Callaghan, Australia; 5 Laboratory of Virology, National Institute of Allergy and Infectious Diseases, National Institutes of Health, Hamilton, MT, USA; 6 Chinese Academy of Medical Sciences Oxford Institute, University of Oxford, Oxford, UK

## Abstract

Seasonal booster vaccination is the primary intervention for protection from respiratory viral infections, such as influenza virus or SARS-CoV-2. However, efficacy is often limited because immune exposure to prior strains impairs development of new responses. In this study, we sought to determine how this issue could be overcome in a mouse model of heterologous immunization against WT and omicron strains of SARS-CoV-2. Intranasal booster immunization circumvented the shortcomings of intramuscular immunization, resulting in superior systemic and mucosal T and B cell immunity and better viral control following SARS-CoV-2 challenge in hamsters. Mechanistically, an intranasal omicron booster immunization bypassed deleterious immune imprinting following intramuscular ancestral strain prime, which allowed for induction of de novo lung B cell and antibody responses against the omicron strain. Cross-reactive memory T cells were also efficiently recruited into the lungs. These findings support further testing of mucosal booster vaccines against respiratory viruses, particularly as a means of simultaneously overcoming deleterious immunological imprinting and enhancing mucosal responses.

## Teaser

Bissett et al. demonstrate that mucosal delivery of heterologous omicron vaccine is able to overcome deleterious prime-derived immunological imprinting via multiple mechanisms, with intranasal boosting responses unaffected by suppressive prime-derived antibodies, and cross-reactive memory T cells engaged and recruited to the lungs.

## Graphical Abstract

**Figure:**
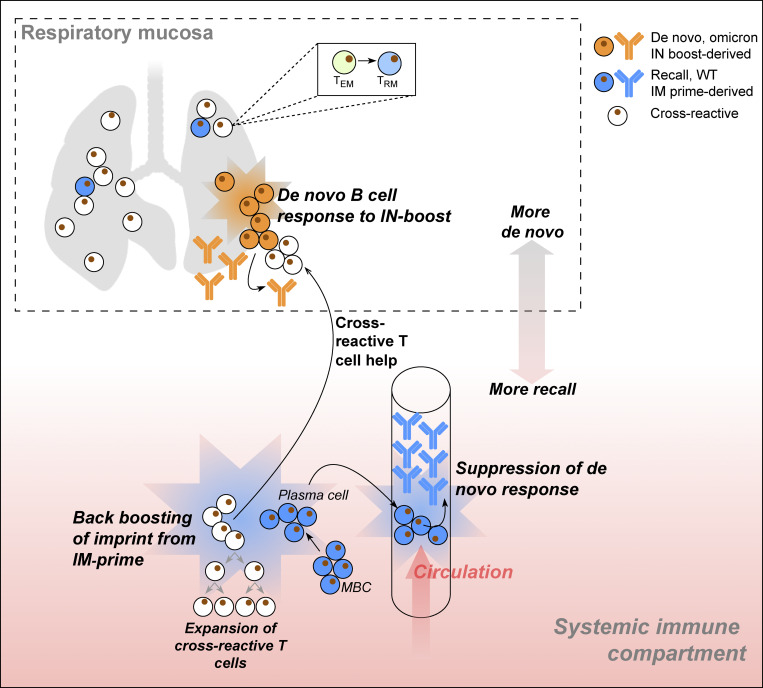

## Introduction

Current vaccines against respiratory viruses such as influenza and SARS-CoV-2 require constant updating as the viruses continually mutate, leading to the evasion of preexisting immunity offered by vaccination and prior infection. Updated booster vaccines are aimed to broaden immunity toward the novel variant. The extensively mutated SARS-CoV-2 variant omicron, for example, has evaded the immunity offered by original, wild-type (WT) spike–encoding SARS-CoV-2 vaccines and previous infection with ancestral virus. This has led to increased incidences of breakthrough infection within the global population. Omicron infections have generally elicited attenuated forms of COVID-19 disease, likely through a combination of preexisting cross-reactive non-neutralizing responses and T cell immunity in the host (Halfmann et al., 2022; Petrone et al., 2023; van Doremalen et al., 2022; Wolter et al., 2022), as well as innate changes to the virus that have resulted in its lower pathogenicity and greater tropism to the upper respiratory tract when compared to earlier variants (Meng et al., 2022). However, many individuals remain vulnerable to more severe disease, and omicron variants are still highly infectious (Bálint et al., 2022; Breznik et al., 2023; Evans et al., 2023; Kahn et al., 2022; Silva et al., 2023; van Kessel et al., 2022).

Although preexisting immunity derived from WT spike vaccination has been linked to reduced disease severity following omicron infection (Buchan et al., 2022; Paul et al., 2023), studies have also revealed this immunity may be restricting immunogenicity to the variant booster vaccines that are administered intramuscularly (IM), via the process of immunological imprinting (Collier et al., 2023; Tortorici et al., 2024; Wang et al., 2023a; Yisimayi et al., 2024; Zaeck et al., 2024). Immunological imprinting describes the influence preexisting immunity (imprint) to an antigen can have on subsequent immune responses to related antigens. The concepts of imprinting termed “negative interference” and “primary addiction” are phenomena that refer to the negative impact of preexisting cross-reactive antibodies on secondary immune responses, and the propensity of the immune response to rely on the primary cohort of memory B cells (MBC) to the detriment of stimulating naive B cell clones, respectively (Schiepers et al., 2023; Zhang et al., 2019). This reactivation of MBC has also been referred to as “back boosting,” and results in the recall of initial antibody responses (Rijkers and van Overveld, 2021). These phenomena can result in the suppression of de novo immune responses. The clinical outcome of primary addiction and negative interference can be lessened protection and/or enhanced infection with the latter pathogen, or the failure to mount an effective immune response to a vaccine (Kim et al., 2009; Zhang et al., 2019).

Administering SARS-CoV-2 variant boosters via alternative routes such as the mucosal route, in what is commonly referred to as a “prime-pull” strategy, may offer a means to overcome imprinting, augment immunogenicity, and critically generate responses at the site of infection (Shin and Iwasaki, 2012). The IM route of vaccination is unable to stimulate the respiratory mucosal compartment as effectively as mucosal routes of vaccination, and strong respiratory mucosal immunity has been associated with enhanced protection against SARS-CoV-2 infection in nonhuman primate and murine studies (Koolaparambil Mukesh et al., 2025; McMahan et al., 2024; Tang et al., 2022; van Doremalen et al., 2021). Notably, lung tissue-resident memory CD8^+^ T cells (T_RM_) and mucosal IgA induced by mucosal vaccination facilitate rapid clearance of infected cells and neutralization of virus, respectively, at the primary site of viral entry and infection (Koolaparambil Mukesh et al., 2025; Lavelle and Ward, 2022; Park et al., 2024).

While the inherent benefit of mucosal delivery of vaccine to induce immunity within the lung has been established, such a vaccine strategy has not been extensively explored and compared with standard IM delivery within the context of deleterious antigenic imprinting. Several preclinical studies have found that an adenovirus vector expressing a heterologous omicron strain SARS-CoV-2 spike antigen when given as a mucosal boosting vaccine induces both robust mucosal IgA and IgG and systemic IgG specific for the omicron strain, which was associated with improved protection from infection (Gagne et al., 2024; McMahan et al., 2024; Xing et al., 2024), thus suggesting that mucosal boosting might simultaneously induce desirable mucosal immunity and also bypass a detrimental imprint toward priming antigens. However, a mechanistic understanding of these promising observations is lacking. Exploration of this alternative route of delivery is warranted given the potential impact it may have on enhancing protection against respiratory diseases such as COVID-19 and influenza.

In this study, we examined heterologous mucosal boosting using omicron and WT spike vaccines. IM boosting with a heterologous omicron vaccine induced minimal omicron-specific antibody immunity. In contrast, we demonstrated that intranasal (IN) administration of omicron vaccine as a booster in mice induced strong omicron-specific and cross-reactive responses both locally in the mucosa and systemically, which afforded superior protection following SARS-CoV-2 omicron BA.1 challenge. Multiple distinct mechanisms were then attributed to the stronger humoral and cellular responses observed following IN boost: the bypassing of the suppressive antibody imprint induced by priming, a de novo–type mucosal B cell response to omicron antigen, and the recall and homing of cross-reactive T cells derived from IM WT vaccination to the lungs.

## Results

### Heterologous mucosal boosting with an omicron vaccine elicits a strong local mucosal and systemic antibody response, and lung T cell response

**Figure 1.**
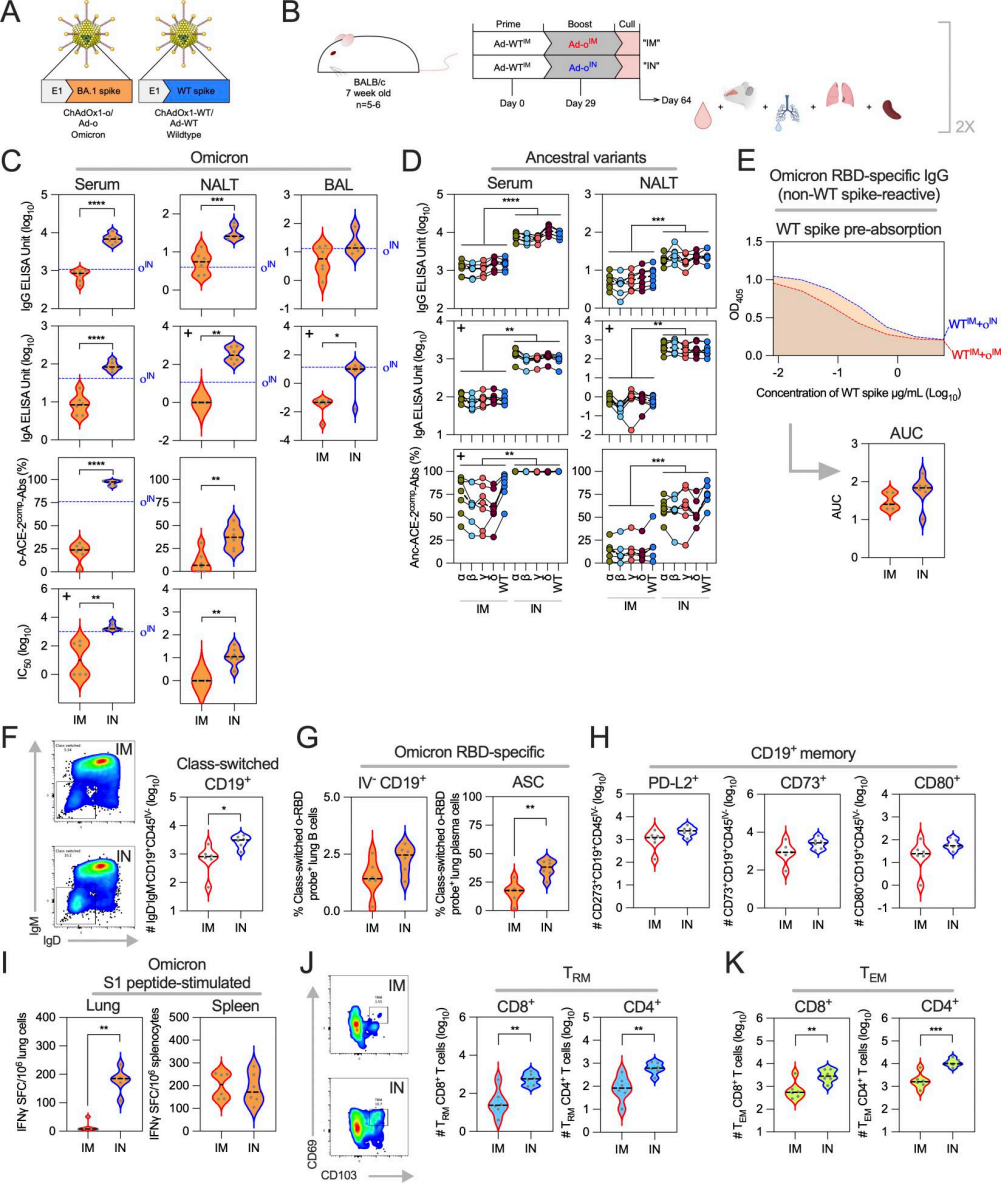
**Heterologous mucosal boosting with an omicron vaccine elicits a strong local mucosal and systemic antibody response, and lung T cell response. (A)** Monovalent ChAdOx1 adenovirus vaccines encoding omicron BA.1 spike (“Ad-o”) or original WT spike (“Ad-WT”) sequences. **(B)** Vaccination schedule for the comparison of heterologous IM or IN boosting of omicron vaccine in WT vaccine–primed mice. 5 wk after boost, sera, NALT, BALF, lungs, and spleens were collected for analyses. **(C)** Omicron-specific antibody responses in the serum, NALT, and BAL fluid. Levels of total omicron spike–specific IgG and IgA were measured by standardized ELISA and presented as log_10_ ELISA units. ACE-2–competing omicron S1-specific antibodies (o-ACE2^comp^-Abs) were measured by Luminex assay and presented as % ACE-2 competition, which was calculated by using the reduction in measured binding compared with a negative internal control. Pseudoneutralization of omicron spike–expressing lentivirus (o-NAbs) was presented as log_10_ IC_50_, which was calculated from sample titration curves. Group median responses following one Ad-o^IN^ prime were represented on graphs as a dashed blue line, with complete data in Fig. S1. **(D)** Responses to earlier SARS-CoV-2 “ancestral” variants alpha (α), beta (β), gamma (γ), delta (δ), and WT in serum and NALT fluid. The dashed black line on plots represents the group median response. **(E)** Levels of non–cross-reactive, o-RBD–specific IgG in sera following IM and IN boosting, as measured through WT spike preabsorption (depletion) assay. The median o-RBD IgG levels in samples that were preincubated with a range of WT spike concentrations is shown at the top of the panel. The AUC values are shown on the violin plot positioned below. **(F)** Total number (log_10_) of class-switched lung B cells (IgD^−^IgM^−^CD19^+^CD45^IV−^) as measured by flow cytometry. **(G)** Frequencies of o-RBD probe–specific lung PCs (CD19^−^CD138^+^IgD^−^IgM^−^o-RBD^+^) and o-RBD probe–specific lung-resident B cells (CD45^IV−^CD19^+^IgD^−^IgM^−^o-RBD^+^). **(H)** Total number (log_10_) of PD-L2-, CD73-, and CD80-expressing lung B cells (CD19^+^CD45^IV−^). **(I)** Frequency of lung cells and splenocytes that released detectable IFNγ following stimulation with omicron S1 peptides (antigen-specific), measured by IFNγ ELISpot assay. **(J)** Total number (log_10_) of lung-resident memory CD8^+^ (CD45^IV−^CD69^+^CD103^+^CD62L^−^CD44^+^) and CD4^+^ T cells (CD45^IV−^CD69^+^CD62L^−^CD44^+^). **(K)** Total number (log_10_) of lung CD8^+^ and CD4^+^ T_EM_ (CD62L^−^CD44^+^CD127^+^). Statistically significant differences between groups in all figures were determined through parametric *t* tests or nonparametric Mann–Whitney tests (*P < 0.05, **P < 0.01, ***P < 0.001); when data did not follow a normal distribution, a + was added to the left corner of the graph. On violin plots, the dashed black line represents the group median and dots represent individual mice. The results in this figure are representative of two independent vaccination experiment repeats (repeat displayed in figure: *n* = 6 per group, and independent repeat: *n* = 5 per group). AUC, area under the curve.

The goal of heterologous vaccination is to broaden responses against novel variants. The administration of omicron booster vaccine via the mucosal IN route, as opposed to standard IM route, was evaluated in mice as a strategy to enhance vaccine immunogenicity. To compare IM and IN boosting, two monovalent SARS-CoV-2 viral vector vaccines encoding the original Wuhan “wild type” (ChAdOx1 nCoV-19, which we have abbreviated to Ad-WT in this study for simplicity) or omicron (ChAdOx1-o, abbreviated Ad-o) full-length spike protein were used. Ad-o and Ad-WT were constructed in a similar fashion; the sequence encoding original WT (Ad-WT) or omicron BA.1 spike (Ad-o) antigen was inserted into the ChAdOx1 backbone, replacing the E1 region (Fig. 1 A). Mice were first vaccinated with Ad-WT IM and then boosted with either Ad-o IM (Ad-WT^IM^+Ad-o^IM^) or IN (Ad-WT^IM^+Ad-o^IN^) 4 wk later, with systemic (spleens and sera) and mucosal (lungs, respiratory fluids) immunogenicity measured a further 5 wk later (day 64 after prime; Fig. 1 B).

ELISA was used to quantify omicron spike–binding IgG and IgA levels in the serum and locally in the respiratory nasal-associated lymphoid tissue (NALT) fluid and bronchoalveolar lavage (BAL) fluid (Fig. 1 C). IN boosting with Ad-o resulted in higher levels of omicron spike–specific IgG in sera and in NALT fluid (P = 0.0001 and P = 0.0002, respectively) and BAL fluid (albeit not statistically significant) compared with IgG levels after IM boosting (Fig. 1 C). Increased IgA responses across immune compartments were also observed following IN boosting, in sera, NALT, and BAL fluid (P ≤ 0.0001, P = 0.0022, and P = 0.0411, respectively) (Fig. 1 C).

**Figure S1.**
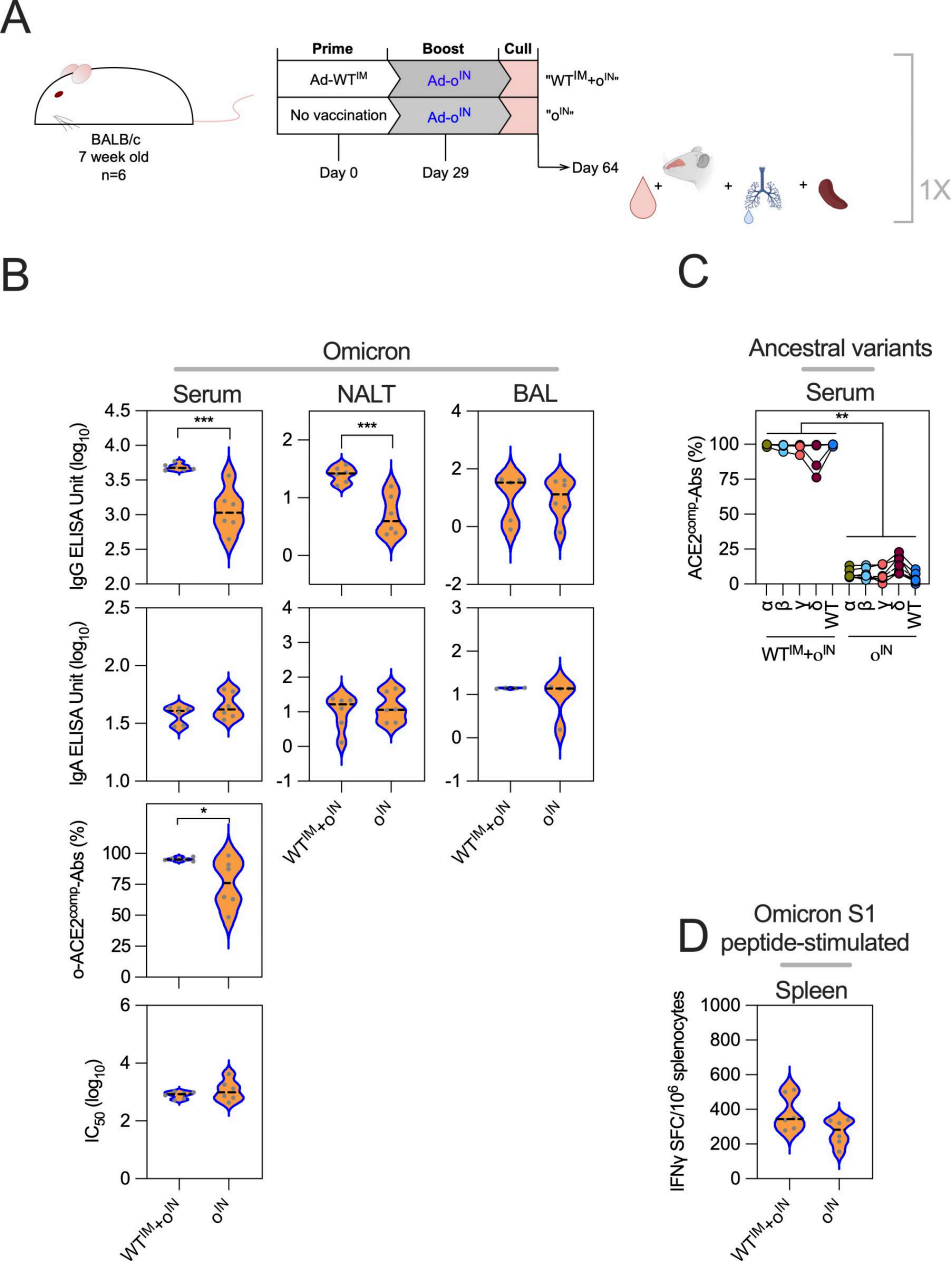
**Ad-WT** ^**IM**^ **+Ad-o** ^**IN**^ **vs. Ad-o** ^**IN**^ **. (A)** Vaccination schedule for the comparison of prime-boost Ad-WT^IM^+Ad-ο^IN^ regimen and prime-only Ad-ο^IN^ regimen. 5 wk after boost, sera, NALT, and BAL fluid were collected. **(B)** Omicron-specific antibody responses in the serum, NALT, and BAL fluid. Levels of total omicron spike–specific IgG and IgA were measured by standardized ELISA and presented as log_10_ ELISA units. ACE-2–competing omicron S1-specific antibodies (o-ACE2^comp^-Abs) were measured by Luminex assay and presented as % competition, which was calculated by using the reduction in measured binding compared with a negative internal control. Pseudoneutralization of omicron spike–expressing lentivirus (o-NAbs) was presented as log_10_ IC_50_, which was calculated from titrated sample curves. **(C)** Responses to earlier ancestral SARS-CoV-2 ancestral variants alpha (α), beta (β), gamma (γ), delta (δ), and WT in serum. The dashed black line on plots represents the group median response. **(D)** IFNγ release by splenocytes stimulated with omicron S1 peptides, measured by IFNγ ELISpot assay. For all data, *P < 0.05, **P < 0.01, ***P < 0.001. To compare between groups, parametric *t* tests were performed. On violin plots, the dashed black line represents the group median response and dots represent individual mice. This experiment was completed once (*n* = 6 per group).

A Luminex ACE-2 competition assay and in-house lentivirus pseudoneutralization assay assessed anti-omicron neutralizing capacity of sera and respiratory fluids; results in each assay were shown to correlate with each other in previously completed correlations that were not included in this study (Fig. 1 C). Higher levels of omicron spike–specific antibodies capable of blocking ACE-2 from binding to spike (o-ACE2^comp^-Abs) were observed in sera (P ≤ 0.0001) and NALT fluid (P = 0.004) following IN boosting of Ad-o compared with IM boosting (Fig. 1 C). IN boosting also induced higher levels of omicron pseudotyped virus-neutralizing antibodies (o-NAbs) compared with IM boosting in sera and NALT (P = 0.0022) (Fig. 1 C). As a control, responses in sera and respiratory fluids following IN boosting were either comparable with or higher than the median response following prime-only Ad-o^IN^ (dashed blue line, Fig. 1 C, complete data set in Fig. S1); this confirms that the IM prime had an enhancing and not deleterious effect on the induction of omicron-targeting antibodies by IN boosting.

We also assessed whether humoral responses to ancestral variants were elicited following boosting with Ad-o, and whether the magnitude of these responses differed following IM or IN boosting (Fig. 1 D). Following IN boosting, elevated levels of both IgG and IgA against all tested ancestral variants were observed in both sera and NALT fluid compared with levels following IM boosting (P ≤ 0.0001 and P = 0.0006, for sera and NALT IgG, respectively, and P = 0.0022 for sera and NALT IgA) (Fig. 1 D). Ancestral variant-binding antibodies also strongly inhibited ACE-2 binding (Anc-ACE2^comp^-Abs) in the Luminex assay (Fig. 1 D). Collectively, administering Ad-o as an IN boost resulted in higher levels of cross-reactive response to ancestral variants both in sera and in NALT, compared with IM administration.

Given the high levels of cross-reactive antibodies observed after IN boost, a WT spike preadsorption assay was completed similar to that described by Pušnik et al. (2024). Samples were normalized for o-RBD-specific antibody titer and preincubated with increasing concentrations of WT spike, prior to the analysis of binding to o-RBD by ELISA. In doing so, the levels of IgG that were specific for distinct, mutated epitopes of o-RBD could be measured. Omicron-binding antibodies induced by IN Ad-o boost were depleted in this competition assay nearly to the level seen following IM boost (Fig. 1 E), suggesting an induction of broadly cross-reactive responses in serum. Collectively, administering Ad-o as an IN boost resulted in higher levels of cross-reactive response to both omicron and ancestral variants both in sera and in NALT, compared with IM administration.

**Figure S2.**
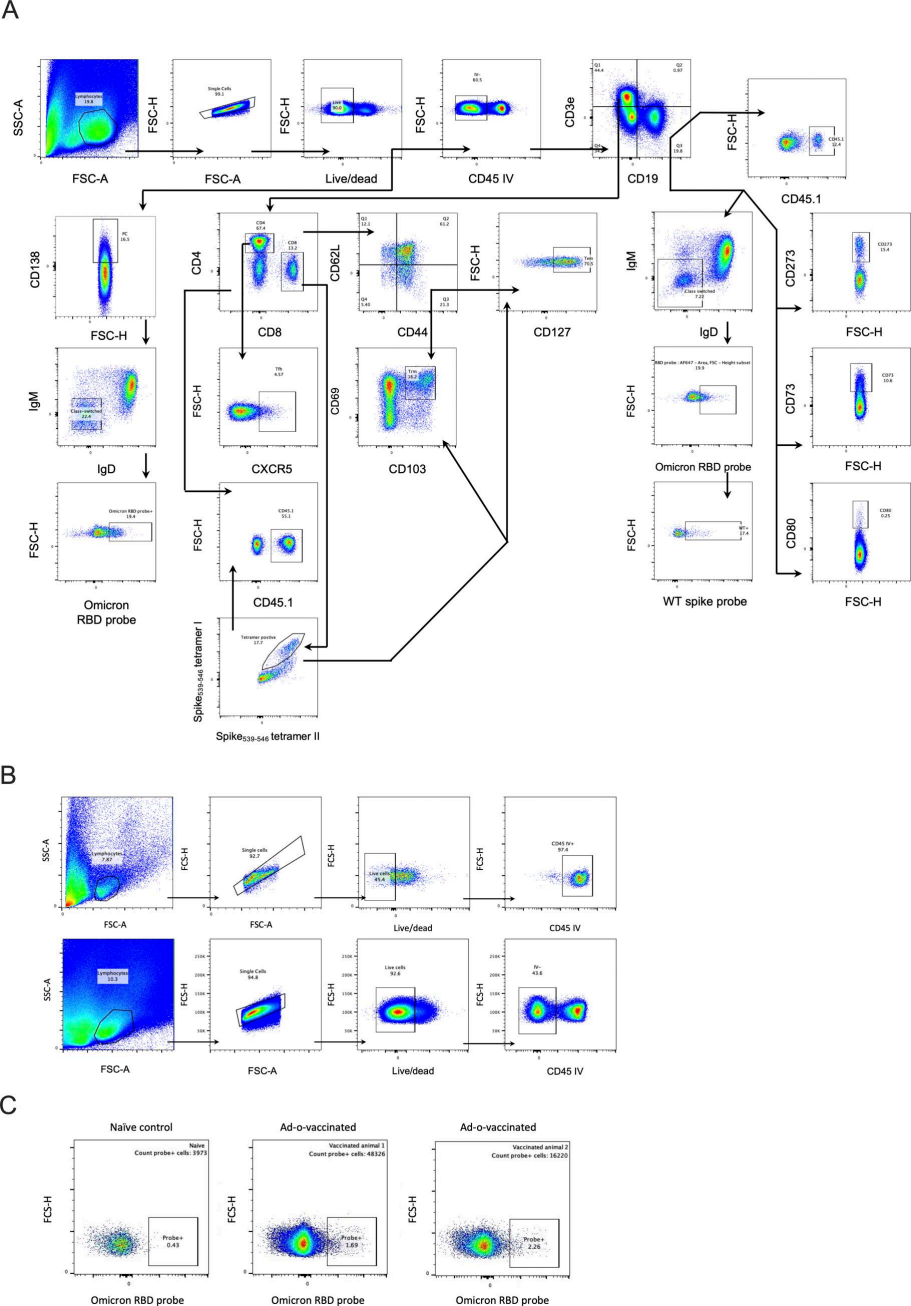
**Flow cytometry gating strategies. (A)** Gating strategy for experiments featured in Figs. 1, 2, and 9. **(B)** Staining of circulatory leukocytes through intravenous injection of anti-CD45 antibody. Example comparison of the relative frequencies of circulatory leukocytes in a PBMC sample and lung sample. **(C)** Example plots from the experiment in Fig. 1 of the staining of B cells using an o-RBD probe. Comparison of naive (negative control) and two positive, vaccinated lung samples. PBMC, peripheral blood mononuclear cell.

Cellular responses following IN or IM boosting were assessed by flow cytometry (Fig. S2), with intravenous anti-CD45 antibody labeling to exclude circulatory B and T cells from the measured lung-resident B and T cell populations. IN-boosted mice had greater numbers of class-switched lung B cells (CD45^IV-^CD19^+^IgD^−^IgM^−^) compared with IM-boosted mice (P = 0.0174, Fig. 1 F). IN-boosted mice also had greater frequencies of class-switched o-RBD–specific lung-resident B cells (defined as CD45^IV−^CD19^+^IgD^−^IgM^−^o-RBD^+^, nonsignificant) and o-RBD–specific plasma cells (PCs) (defined as CD19^−^CD138^+^IgD^−^IgM^−^o-RBD^+^, P = 0.0014) compared with IM-boosted mice (Fig. 1 G). Greater numbers of lung B cells (CD45^IV−^CD19^+^) expressing common memory markers PD-L2, CD73, and CD80 (all nonstatistically significant difference) were observed following IN boosting compared with that following IM boosting (Fig. 1 H) (Tomayko et al., 2010).

We next examined the impact of IN boosting on the T cell responses. Omicron-specific IFNγ T cell responses in the spleen and lungs were measured using an ELISpot assay (Fig. 1 I). Cells were stimulated with peptides spanning the S1 domain of spike that contained omicron-specific mutations (Table S1). A greater number of S1-specific lung cells were observed in IN-boosted mice compared with IM-boosted (P = 0.0022, Fig. 1 I), while in the spleen, comparable numbers of IFNγ-producing cells were measured (Fig. 1 I). Lung T_RM_ were defined as CD45^IV-^CD69^+^CD103^+^CD62L^−^CD44^+^ when CD8^+^ and CD45^IV−^CD69^+^CD62L^−^CD44^+^ when CD4^+^. CD8^+^ and CD4^+^ effector memory T cells (T_EM_) were both defined as CD62L^−^CD44^+^CD127^+^. Greater numbers of CD8^+^ and CD4^+^ lung T_RM_ (CD8^+^, P = 0.0030; CD4^+^, P = 0.0046, Fig. 1 J) and T_EM_ (CD8^+^ P = 0.0089, CD4^+^ P = 0.0004, Fig. 1 K) were observed by IN boosting when compared to IM boosting.

Collectively, IN booster administration of Ad-o elicited stronger anti-omicron responses in the blood and respiratory fluids, compared with IM booster administration. Ad-WT^IM^+Ad-o^IN^ vaccination also resulted in broad antibody responses to ancestral variants in the blood and NALT. IN administration of Ad-o resulted in enhanced lung T and B cell responses compared with when administered IM, with noninferiority of systemic T cell responses.

### Mucosal immunity is measurable months after mucosal boosting with an omicron vaccine

**Figure 2.**
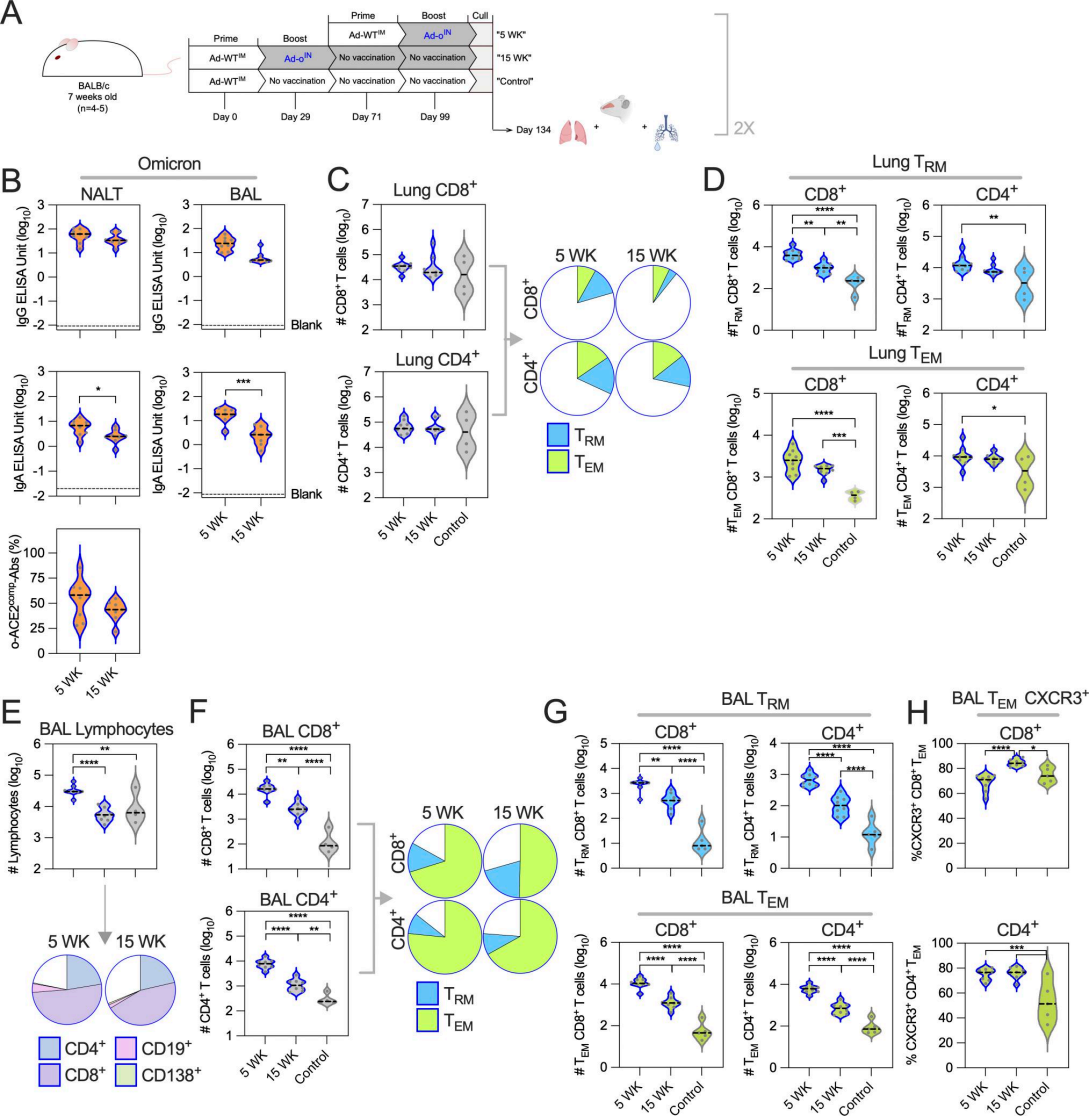
**Respiratory mucosal immunity is still elevated at 3 months following IN boosting but wanes with time. (A)** Vaccination schedule comparison of immune responses at 5 wk or 15 wk after an Ad-ο^IN^ boost, with an IM prime-only group as a comparison. **(B)** Omicron-specific antibody responses in the serum, NALT, and BAL fluid. Levels of total omicron spike–specific IgG and IgA were measured by standardized ELISA and presented as log_10_ ELISA units. ACE-2–competing omicron S1-specific antibodies (o-ACE2^comp^-Abs) were measured by Luminex assay and presented as % ACE-2 competition, which was calculated by using the reduction in measured binding compared with a negative internal control. ELISA titer limit of detection (“Blank”) is noted for reference. **(C)** Total number (log_10_) of CD8^+^ and CD4^+^ T cells in the lung (left) and proportion (group median) of T_RM_^+^ (CD8^+^: CD69^+^CD103^+^CD62L^−^CD44^+^ and CD4^+^: CD69^+^CD62L^−^CD44^+^) and T_EM_^+^ (CD62L^−^CD44^+^CD127^+^ for both CD4^+^ and CD8^+^) cells (right). **(D)** Total number (log_10_) of lung-resident memory CD8^+^ and CD4^+^ T cells (CD8^+^: CD69^+^CD103^+^CD62L^−^CD44^+^ and CD4^+^: CD69^+^CD62L^−^CD44^+^) and effector memory CD8^+^ and CD4^+^ T cells (CD62L^−^CD44^+^CD127^+^). **(E)** Total number (log_10_) of lymphocytes in BAL fluid (top) and proportion (group median) of CD8^+^ and CD4^+^ T cells, CD19^+^ B cells, and CD138^+^CD19^−^ PCs (bottom). **(F)** Total number (log_10_) of CD8^+^ and CD4^+^ T cells in the BAL fluid (left) and proportion (group median) of T_RM_^+^ and T_EM_^+^ cells (defined as in C) (right). **(G)** Total number (log_10_) of BAL-resident memory CD8^+^ and CD4^+^ T cells and effector memory CD8^+^ and CD4^+^ T cells (defined as in C). **(H)** Proportion of BAL effector memory CD8^+^ and CD4^+^ T cells expressing CXCR3. For all data, *P < 0.05, **P < 0.01, ***P < 0.001, ****P ≤ 0.0001. Parametric *t* tests or one-way ANOVA tests with multiple comparison testing were performed. On violin plots, the dashed black line represents the group median response and dots represent individual mice. The data shown in this figure are pooled from two independent experiments where test groups “5 WK” and “15 WK” were included (*n* = 4–5 per group). The “control” test group (*n* = 5) was uniquely included in one of the independent experiments.

Lung mucosal immunity is known to wane after exposure (Caminschi et al., 2019; Pizzolla et al., 2017; Slütter et al., 2017), so we sought to determine the durability of responses induced by IN Ad-o boosting. Mice were vaccinated Ad-WT^IM^+Ad-o^IN^, and NALT, BAL, and lung tissue were collected after either five or 15 wk (Fig. 2 A). As an additional control, a group of mice were also primed Ad-WT^IM^ at the same time as the 15 wk group, but not boosted; these mice were culled at the same time as the two experimental groups. Over the 15-wk period, omicron-specific IgG in NALT and BAL fluid did not significantly decrease, nor did o-ACE2^comp^-Abs in the NALT fluid, although waning was qualitatively observed (Fig. 2 B). In contrast, omicron-specific IgA levels significantly declined in both NALT and BAL fluid (P = 0.02 and P = 0.0002, respectively), but were still substantially above the limit of detection (Fig. 2 B).

We also examined the persistence of cellular responses over the 15-wk period in the mucosa. There was no difference in the overall number of CD8^+^ or CD4^+^ T cells in the lungs between 5 and 15 wk after boost (Fig. 2 C). The CD8^+^ T_RM_ cell, CD4^+^ T_RM_ cell, CD8^+^ T_EM_ cell, and CD4^+^ T_EM_ cell populations were all significantly elevated at 5 wk after boost, and there was a qualitative contraction of these responses by 15 wk, which was significant only in the CD8^+^ T_RM_ cell population (P = 0.0004; Fig. 2 D). In BAL fluid, the overall number of lymphocytes induced following IN boost decreased from 5 to 15 wk (P < 0.0001) to levels comparable to mice that had not received the IN boost, though the relative frequencies of lymphocyte subsets remained generally similar (Fig. 2 E). The number of BAL CD8^+^ or CD4^+^ T cells significantly waned by 15 wk, but were still significantly elevated compared with mice, which did not receive an IN boost (P < 0.0001 and P = 0.001, respectively; Fig. 2 F). These were predominantly T_RM_ and T_EM_ cells (Fig. 2 F), and while these cells decreased in frequency from 5 to 15 wk after boost, they were significantly more abundant in the BAL at 15 wk compared with mice that had not received the IN boost (P < 0.0001; Fig. 2 G). The CD8^+^ and CD4^+^ T_EM_ cells maintained high expression of CXCR3 across the time points, a marker associated with cell retention and survival within the lungs (Fig. 2 H). Collectively, while both humoral and cellular mucosal immunity waned over a 15-wk period, these responses were still significantly elevated suggesting durable protection.

### Mucosal boosting of omicron vaccine protects against viral replication in the respiratory tract of Syrian hamsters

**Figure S3.**
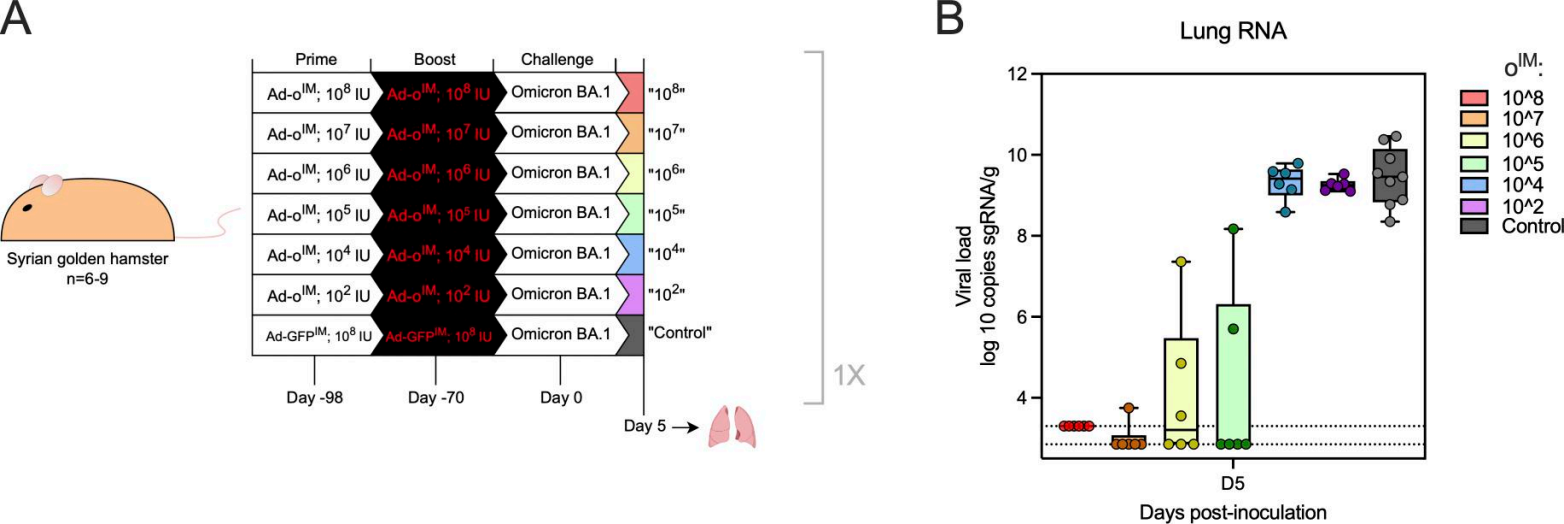
**Determination of partially protective dose of Ad-omicron in hamsters. (A)** Syrian golden hamsters were vaccinated with log-fold dilutions of Ad-ο^IM^ in a homologous prime-boost regimen from 10^2^ to 10^8^ infectious units (IU) followed by a challenge 70 days later with SARS-CoV-2 omicron BA.1. **(B)** Viral load in lung tissue as determined by PCR quantitation of sgRNA. Dots represent individual animals with box plots denoting median ± IQR and whiskers at min to max. This experiment was completed once (Ad-GFP^IM^ control group, *n* = 9; Ad-o^IM^ test group, *n* = 6). sgRNA, subgenomic RNA.

Next, we investigated whether the observed enhanced immunogenicity described in the studies above would translate to protection against SARS-CoV-2 in an established animal model. Initially, we defined a suboptimal vaccine dose that was not fully protective, so relative efficacy of IM versus IN boosting could be assessed effectively. Syrian hamsters were vaccinated at 0 and 4 wk with Ad-o^IM^ using 10-fold serial dilutions of the vaccine, ranging from 10^8^ infectious units (IU)/animal to 10^2^ IU/animal (Fig. S3 A). At week 10, animals were challenged with a total dose of 10^4^ TCID_50_ of the homologous virus omicron variant BA.1. Vaccine doses at 10^4^ IU/animal or lower were found to not reduce viral load in the lungs, doses from 10^5^ to 10^7^ IU/animal resulted in variable reduction in lung viral loads, and a dose of 10^8^ IU/animal resulted in no detectable viral loads (Fig. S3 B).

**Figure 3.**
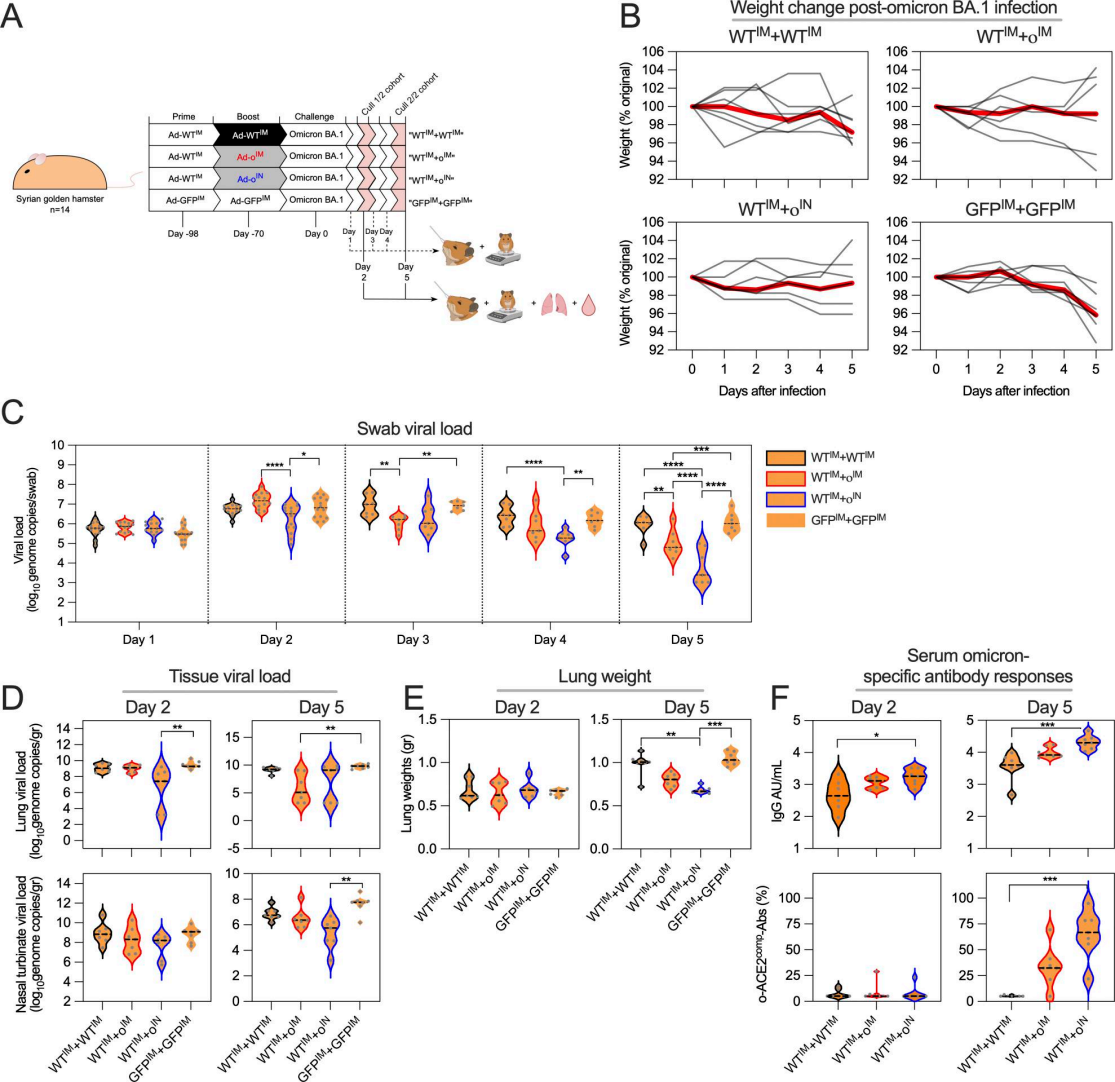
**IN boosting affords superior protection following SARS-CoV-2 omicron BA.1 challenge in a hamster model. (A)** Vaccination and infectious challenge schedule examining the protective efficacy of Ad-WT^IM^ versus Ad-ο^IM^ versus Ad-ο^IN^ boost against a SARS-CoV-2 omicron BA.1 challenge in Syrian golden hamsters. Hamsters were followed for 5 days following challenge, with subgroups killed on day 2 and 5 after challenge to assess tissue responses. **(B)** Weight change kinetics following BA.1 challenge. Each line represents an individual animal, and the red line is group median. **(C)** Viral load assessed by PCR of nasal swabs. **(D)** Viral load assessed by PCR of lung homogenates or nasal washes. **(E)** Lung weight. **(F)** Omicron-specific IgG antibody responses in serum and ACE-2–competing omicron S1–specific antibodies (o-ACE2^comp^-Abs). For all data, *P < 0.05, **P < 0.01, ***P < 0.001, ****P ≤ 0.0001. Parametric one-way ANOVA tests with multiple comparison testing were performed. On violin plots, the dashed black line represents the group median response and dots represent individual mice. Data shown are from one experiment (*n* = 14).

We next conducted a challenge study with a dose of 10^5^ IU/animal comparing IM versus IN boosting (Fig. 3 A). All animals were primed IM with Ad-WT. 4 wk later, animals were boosted with either Ad-WT^IM^, Ad-o^IM^, or Ad-o^IN^. As a control, animals were vaccinated with ChAdOx1-GFP, IM, at weeks 0 and 4. At week 10, animals were challenged with omicron BA.1 variant, as described above. All vaccinated hamsters had minimal weight loss following challenge (Fig. 3 B). Oropharyngeal swabs were collected daily to assess virus shedding. Shedding of viral RNA was significantly reduced in the Ad-o^IN^ group as early as 2 days after challenge with significantly lower viral loads 5 days after challenge as compared to all other groups (Fig. 3 C). Viral RNA levels in the nasal turbinates 5 days after inoculation were also significantly reduced only in group Ad-o^IN^ compared with controls (Fig. 3 D). Finally, viral load was measured in lung tissues at two and 5 days after inoculation. At 2 days, Ad-o^IN^ animals showed significantly reduced viral loads compared with other groups. However, by 5 days after challenge, both IM and IN Ad-o–vaccinated groups exhibited significantly lower lung viral loads compared with controls, whereas no differences were noted between controls and the Ad-WT^IM^ group. Lung weights, as an indication of edema and cellular influx, were significantly increased 5 days after challenge in unvaccinated controls and hamsters that were boosted IM with Ad-WT compared with the Ad-o^IN^ group (Fig. 3 E). Thus, IN boosting with Ad-o provided superior viral control and minimized pathology.

Serum was collected at necropsy, and BA.1-specific IgG and ACE-2 binding competition titers were assessed. There was a stepwise increase in omicron-binding Ab titers and o-ACE2^comp^-Abs from Ad-WT^IM^ to Ad-o^IM^ to Ad-o^IN^ immunization, which corresponded to the levels of viral control (Fig. 3 F).

In sum, IN omicron booster provided superior viral control following challenge than did an intramuscular boosting vaccine.

### Matching the prime-boost vaccination route dampens omicron-specific antibody responses induced by a heterologous omicron booster vaccine

**Figure 4.**
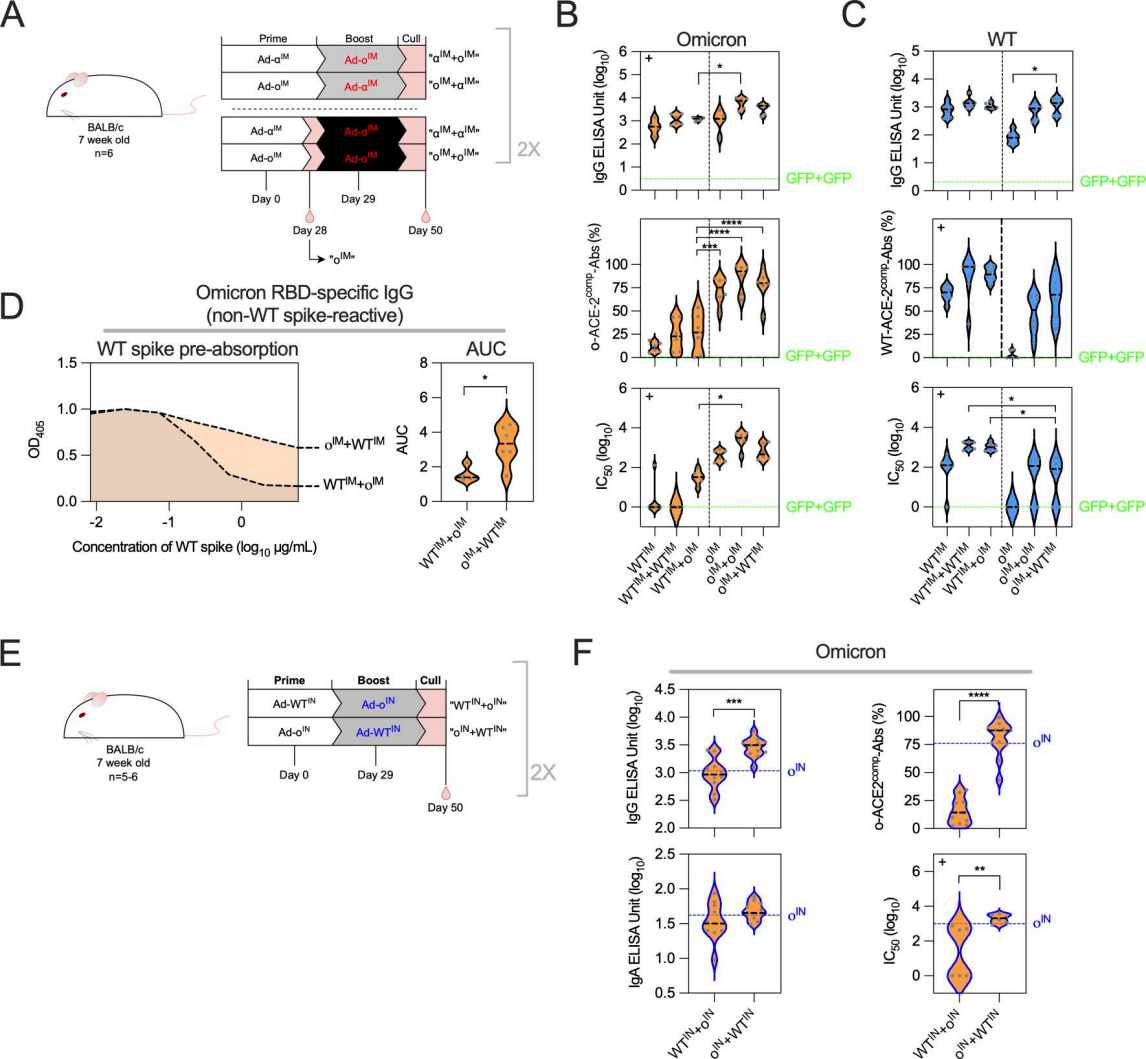
**Matching the prime-boost vaccination route dampens omicron-specific antibody responses induced by a heterologous omicron booster vaccine. (A)** Vaccination schedule for the comparison of heterologous IM prime-boost regimens (Ad-WT^IM^+Ad-ο^IM^ and Ad-ο^IM^+Ad-WT^IM^) and homologous IM prime-boost regimens (Ad-WT^IM^+Ad-WT^IM^ and Ad-ο^IM^+Ad-ο^IM^); heterologous and homologous experiments were performed separately following identical vaccination schedules. **(B–D)** Heterologous vaccination experiment was repeated (*n* = 5–6 per group), with data representative of one repeat experiment (*n* = 6 per group) present in figures (B–D). Sera were collected from mice in the homologous prime-boost regimens 4 wk after prime to measure prime-only responses, and 3 wk after boost for prime-boost regimens. **(B)** Levels of total omicron spike–specific IgG were measured by standardized ELISA and presented as log_10_ ELISA units (EU). ACE-2–competing omicron S1-specific antibodies (o-ACE2^comp^-Abs) were measured by Luminex assay and presented as % ACE-2 competition. Pseudoneutralization of omicron spike–expressing lentivirus (o-NAbs) was presented as log_10_ IC_50_. Median responses of negative control sera from mice vaccinated twice with an irrelevant vaccine (ChAdOx1-GFP) were included as a green dashed line on graphs (GFP+GFP). **(C)** Levels of total WT spike–specific IgG, WT-ACE2^comp^-Abs, and WT-NAbs were measured, presented as in B. **(D)** Levels of non–cross-reactive, o-RBD–specific IgG in sera following Ad-WT^IM^+Ad-ο^IM^ and Ad-ο^IM^+Ad-WT^IM^, as measured through WT spike preabsorption (depletion) assay. The median o-RBD IgG levels in samples that were preincubated with a range of full-length WT spike concentrations are shown on the left. The AUC values are shown on the right. **(E)** Vaccination schedule for the comparison of heterologous IN prime-boost where the order of immunization of Ad-ο^IN^ and Ad-WT^IN^ was reversed. Data are pooled from two independent experiments (*n* = 5–6 per group per experiment). **(F)** Levels of total omicron spike–specific IgG, omicron spike–specific IgA, o-ACE2^comp^-Abs, and o-NAbs were measured, presented as in B. For IgG, ACE2^comp^-Abs and NAbs data in B Ad-WT^IM^+Ad-ο^IM^ were compared against all other groups and (C) Ad-ο^IM^+Ad-WT^IM^ was the comparator. Parametric one-way ANOVA tests were completed when data were normally distributed; otherwise, a nonparametric Kruskal–Wallis test was performed (indicated with a + at the top left of the graph). A parametric *t* test was used to compare groups in D and F, or a nonparametric Mann–Whitney U test was used (indicated with a + at the top left of the graph). For all data, *P < 0.05, **P < 0.01, ***P < 0.001, and ****P ≤ 0.0001. On violin plots, the dashed black line represents the group median and dots represent individual mice. AUC, area under the curve.

To investigate whether the dampened responses following IM administration of an omicron vaccine were a result of immunological imprinting from previous IM Ad-WT vaccination, the immunogenicity of Ad-WT^IM^+Ad-o^IM^ (both doses delivered IM) was compared with the reverse regimen Ad-o^IM^+Ad-WT^IM^ (both doses delivered IM), and homologous and prime-only Ad-WT^IM^ and Ad-o^IM^ IM regimens (Fig. 4 A). Two indications of immunological imprinting could be investigated via serological analysis: firstly, back boosting, as measured in a bias in antibody response toward the prime vaccine antigen, and secondly, suppression of de novo responses to the boost antigen, measured by comparing with levels generated after one prime dose of vaccine.

3 wk after boost, anti-omicron IgG was detected in the sera of all vaccinated mice (Fig. 4 B). Ad-WT^IM^+Ad-o^IM^–vaccinated mice induced lower levels of anti-omicron spike IgG than Ad-o^IM^+Ad-o^IM^- (P = 0.0105) and Ad-o^IM^+Ad-WT^IM^–vaccinated (not significant) mice, but had comparable levels to a single IM dose of Ad-o (Fig. 4 B).

Although anti-omicron spike IgG antibodies were detected after Ad-WT^IM^+Ad-o^IM^ vaccination, these antibodies displayed low levels of omicron neutralization (Fig. 2 B). o-ACE2^comp^-Ab levels following Ad-WT^IM^+Ad-o^IM^ (median 26.94% ACE-2 competition) were equivalent to that following Ad-WT^IM^+Ad-WT^IM^ vaccination (median 22.82% ACE-2 competition) (Fig. 4 B). These o-ACE2^comp^-Ab levels were significantly lower than those measured following Ad-o^IM^+Ad-WT^IM^ (2.97-fold lower; P ≤ 0.0001), Ad-o^IM^+Ad-o^IM^ (3.42-fold lower; P ≤ 0.0001), and even Ad-o^IM^ (2.8-fold lower; P = 0.0001) vaccination (Fig. 4 B). A similar trend was noted with levels of omicron pseudotyped virus neutralization, with Ad-WT^IM^+Ad-o^IM^ vaccination generating lower levels of o-NAbs compared with Ad-o^IM^+Ad-WT^IM^ (1.91-fold lower), Ad-o^IM^+Ad-o^IM^ (2.35-fold lower), and Ad-o^IM^ (1.75-fold lower) (Fig. 4 B). In summary, the priming WT vaccine dose strongly limited the ability of heterologous omicron vaccination to induce o-ACE2^comp^-Abs and o-NAbs.

Anti-WT spike responses (Fig. 4 C) were also assessed with a similar trend to anti-omicron responses observed. The Ad-o^IM^+Ad-WT^IM^ regimen induced detectable anti-WT spike IgG titers of comparable level to cognate Ad-WT^IM^ and Ad-WT^IM^+Ad-WT^IM^ and heterologous Ad-WT^IM^+Ad-o^IM^ regimens (Fig. 4 C). However, Ad-o^IM^+Ad-WT^IM^ vaccination generated lower levels of WT-ACE2^comp^-Abs (1.33-fold lower) and WT-NAbs (1.57-fold lower; P = 0.0272) than Ad-WT^IM^+Ad-o^IM^ (Fig. 4 C). Although slight differences were observed based on the specific antigen, the same trend was seen in both cases: functional antibody responses were biased toward the priming strain regardless of the booster vaccine used.

The presence of omicron-reactive antibodies in Ad-WT^IM^+Ad-o^IM^-vaccinated mice could be due to either cross-reactive antibodies initially induced by Ad-WT^IM^ and back-boosted by Ad-o^IM^, or by induction of de novo omicron-specific responses by Ad-o^IM^. Following Ad-o^IM^+Ad-WT^IM^ vaccination, higher titers of non–cross-reactive, o-RBD-specific IgG were detected in sera compared with Ad-WT^IM^+Ad-o^IM^ vaccination (P = 0.0203; Fig. 4 D). Thus, Ad-WT^IM^+Ad-o^IM^ vaccination primarily induced cross-reactive as opposed to de novo omicron-specific Ab responses.

**Figure S4.**
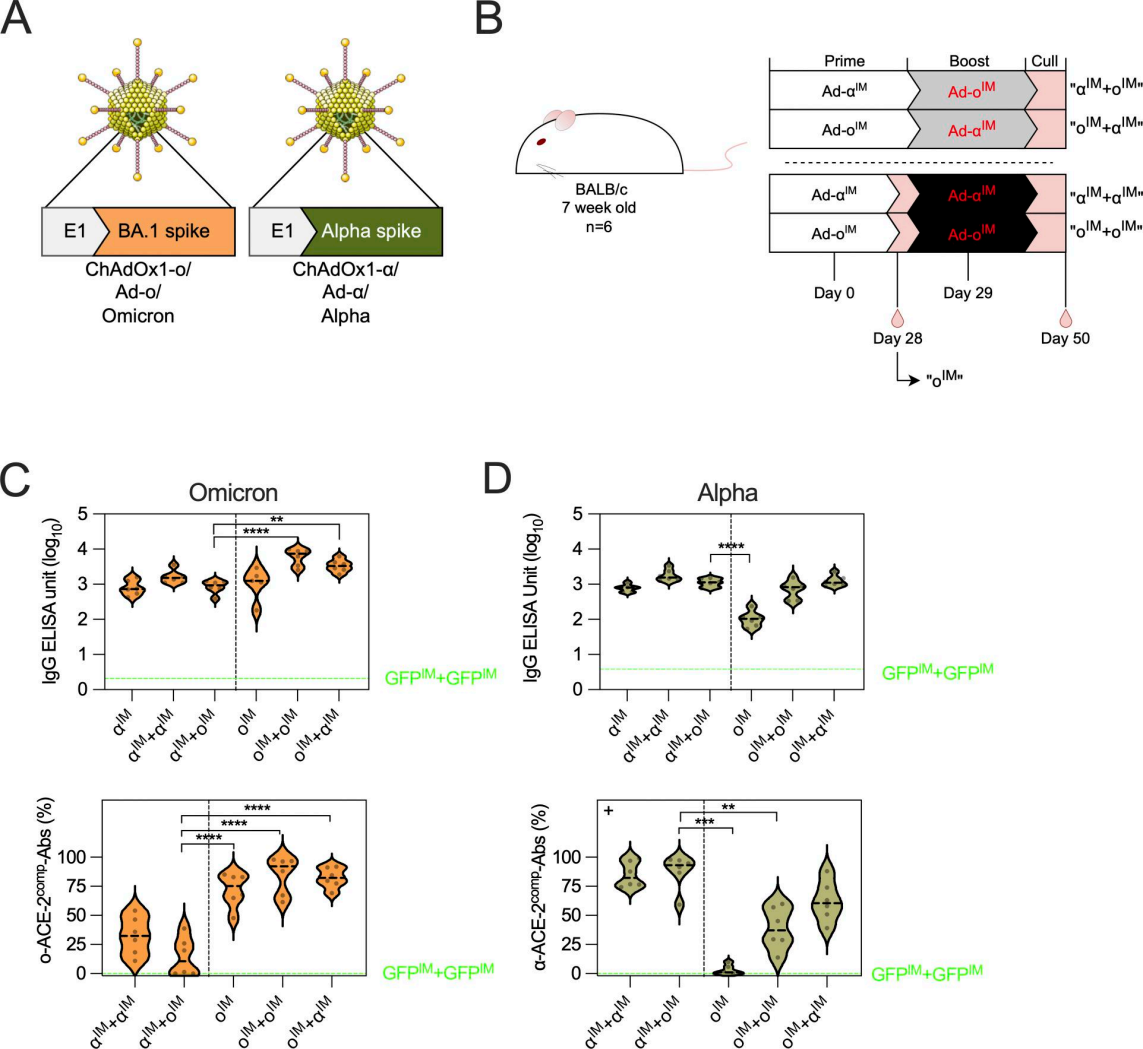
**Heterologous IM boosting of omicron vaccine results in a poor omicron-specific antibody response in alpha spike vaccine–primed mice. (A)** Monovalent ChAdOx1 adenovirus vaccines encoding omicron BA.1 spike (Ad-ο) or alpha spike (“Ad-α”) sequences. **(B)** Vaccination schedule for the comparison of heterologous IM prime-boost regimens (Ad-α^IM^+Ad-ο^IM^ and Ad-ο^IM^+Ad-α^IM^) and homologous IM prime-boost regimens (Ad-α^IM^+Ad-α^IM^ and Ad-ο^IM^+Ad-ο^IM^); homologous and heterologous experiments were performed separately following identical schedules. Sera were collected from mice in the homologous prime-boost regimens 4 wk after prime to measure prime-only responses, and 3 wk after boost for prime-boost regimens. **(C)** Levels of total omicron spike–specific IgG were measured by standardized ELISA and presented as log_10_ ELISA units (EU). ACE-2–competing omicron S1-specific antibodies (o-ACE2^comp^-Abs) were measured by Luminex assay. Median responses of negative control sera from mice vaccinated twice with an irrelevant vaccine (ChAdOx1-GFP) were included as a green dashed line on graphs (GFP^IM^+GFP^IM^). **(D)** Levels of total α spike–specific IgG and α-ACE2^comp^-Abs were measured and presented as in C. For IgG and ACE2^comp^-Abs data in C and D, Ad-α^IM^+Ad-ο^IM^ was compared against all other groups statistically. Parametric one-way ANOVA tests were completed when data were normally distributed; otherwise, a nonparametric Kruskal–Wallis test was performed (indicated with a + at the top left of the graph). For all data, **P < 0.01, ***P < 0.001, and ****P ≤ 0.0001. On violin plots, the dashed black line represents the group median response and dots represent individual mice. This experiment was completed once (*n* = 6 per group).

Collectively, Ad-WT^IM^-priming did not enhance, and sometimes reduced, the immunogenicity of the heterologous IM Ad-o boost, with mice having generated a limited omicron-specific functional humoral response. A similar trend was observed when mice were vaccinated with an alpha spike–encoding vaccine (Ad-α [alpha]) and then boosting with Ad-o^IM^, where limited omicron-specific responses were measured (Fig. S4).

We next sought to determine whether the suboptimal induction of omicron-specific antibodies by IM boost following IM prime was generalizable to alternate vaccination routes. Mice were primed IN with Ad-WT and boosted 4 wk later IN with Ad-o, and this was compared to vaccination with the reverse order (Fig. 4 E). The Ad-WT^IN^+Ad-o^IN^ prime-boost regimen induced significantly reduced serum omicron-specific IgG titers (P < 0.001), o-ACE2^comp^-Ab levels (P < 0.0001), and o-NAb levels (P < 0.01) compared with the reverse Ad-o^IN^+ Ad-WT^IN^ prime-boost regimen (Fig. 4 F). Thus, the priming antigen strongly dictates the breadth of the humoral response elicited by heterologous vaccination when both vaccines are delivered by the same route.

### Extending the prime-boost interval inhibits the efficacy of intramuscular Ad-omicron boost

**Figure 5.**
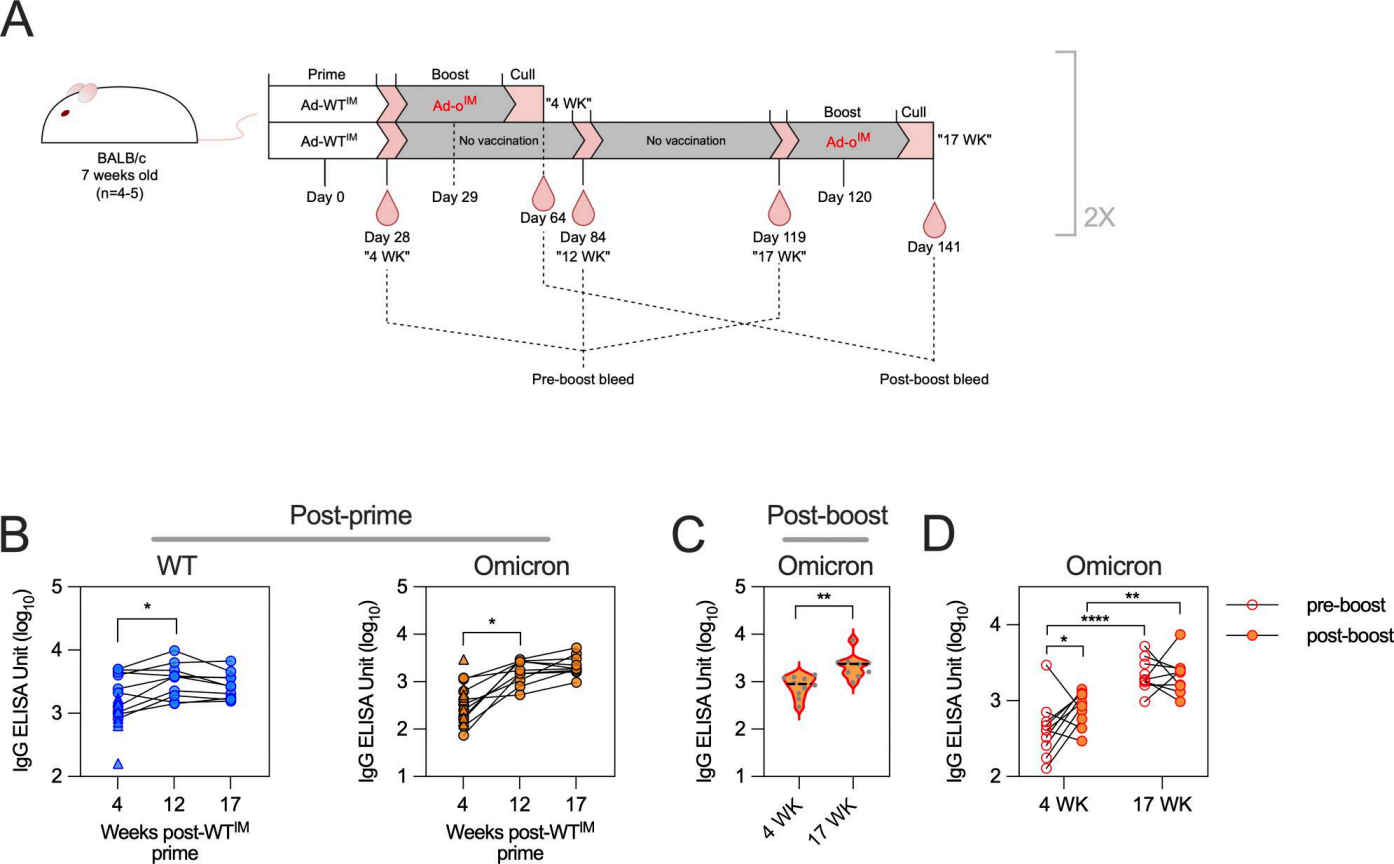
**Extending the prime-boost interval inhibits the efficacy of intramuscular Ad-omicron boost. (A)** Vaccination schedule to vary the interval between prime and boost. Mice were given Ad-WT^IM^+Ad-ο^IM^ with either 4 or 17 wk between the prime and boost. Serum was collected 3–5 wk after the boosting immunization. Data are pooled from two independent experiments (*n* = 4–5 per group per experiment). **(B)** WT and omicron-specific IgG antibody responses in the serum measured by standardized ELISA and presented as log_10_ ELISA units. **(C)** Omicron-specific antibody responses in the serum. Levels of total omicron spike–specific IgG were measured by standardized ELISA and presented as log_10_ ELISA units. **(D)** Omicron-specific antibody responses in the serum before and after Ad-ο^IM^ boosting immunization. Preboost is defined as 1 day before boost vaccination. After boost is defined as 3–5 wk postboost vaccination. Data in B were analyzed by paired one-way ANOVA, in C were analyzed by *t* test, and in D by mixed-effects ANOVA. *P < 0.05, **P < 0.01, ****P < 0.01. On violin plots, the dashed black line represents the group median response and dots represent individual mice.

We hypothesized the limited induction of omicron-specific responses after IM Ad-o boost might be counteracted by increasing the interval between prime and boost, when antibodies against WT spike may have waned. The standard 4-wk interval was compared with an extended 17-wk interval, with responses measured 3 to 5 wk after the boost (Fig. 5 A). Surprisingly, anti-WT and anti-omicron antibody titers continued to increase from 4 to 17 wk after prime (Fig. 5 B). Thus, while anti-omicron IgG titers were significantly higher after boost when a 17-wk interval was used (P < 0.01; Fig. 5 C), this was due only to the higher preboost titers (Fig. 5 D). Indeed, no significant boosting of omicron-specific responses was observed in the 17-wk group, while the 4-wk group had a significant increase in omicron-specific IgG titers (P < 0.05; Fig. 5 D). Thus, preexisting immunity appeared to directly suppress development of omicron-specific responses by an IM Ad-o booster.

### Antibodies from IM WT vaccine priming suppress the latter IM omicron vaccine boost response

**Figure 6.**
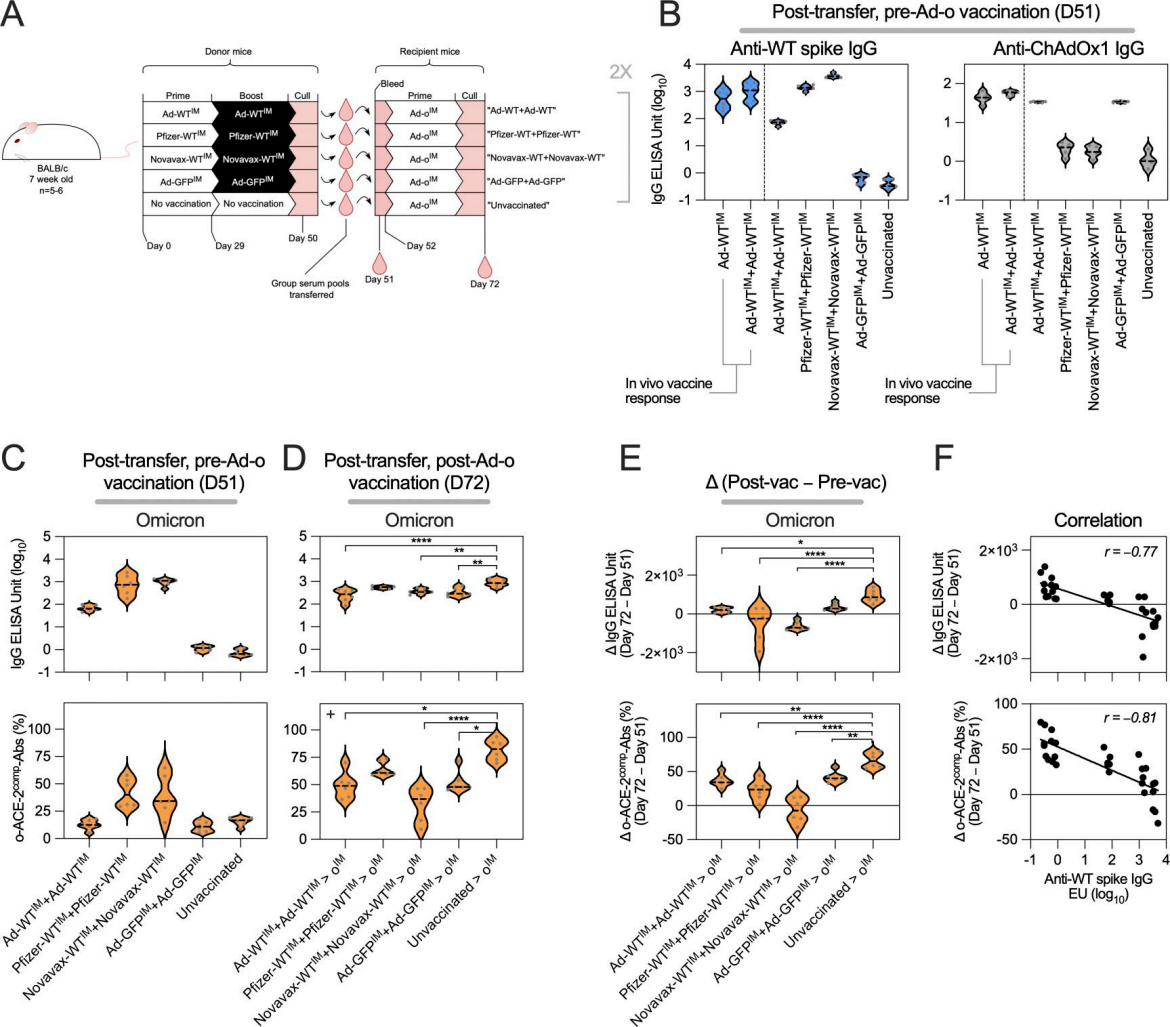
**Antibodies from IM WT vaccine priming suppress the latter IM omicron vaccine boost response. (A)** Vaccination and serum transfer schedule. Donor mice were vaccinated (or left unvaccinated as a negative control), with sera collected and transferred to naive recipient mice. Recipient mice were vaccinated with Ad-ο IM. Sera from recipient mice were collected after transfer of donor sera before vaccination (day 51) and 3 wk after Ad-ο vaccination (day 72). **(B)** Levels of anti-WT spike IgG and anti-ChAdOx1 IgG in recipient mouse sera, after transfer of donor sera, and before Ad-o vaccination (day 51). Reference levels of IgG following an in vivo Ad-WT^IM^ and Ad-WT^IM^+Ad-WT^IM^ are shown. **(C)** Levels of anti-omicron spike IgG and o-ACE2^comp^-Abs in mouse sera, after transfer of donor sera, and before Ad-o vaccination (day 51). **(D)** Levels of anti-omicron spike IgG and o-ACE2^comp^-Abs in mouse sera, after transfer of donor sera, and after Ad-o vaccination (day 72). **(E)** Absolute change (day 72–day 51) in anti-omicron spike IgG and o-ACE2^comp^-Ab levels. **(F)** Correlation of absolute change (day 72–day 51) in anti-omicron spike IgG and o-ACE2^comp^-Ab levels against levels of anti-WT spike IgG transferred (measured on day 51). Data in D were analyzed by one-way ANOVA test on IgG data, and Kruskal–Wallis test on o-ACE2^comp^-Abs data. Data in E were analyzed by one-way ANOVA test with post hoc comparison against mice receiving naive sera as the reference. **(F)** Pearson correlations are shown. For all data, *P < 0.05, **P < 0.01, and ****P ≤ 0.0001. On violin plots, the dashed black line represents the group median and dots represent individual mice. Data are representative of two independent experiments, where experiment 1 (shown) was *n* = 6 per group and experiment 2 was *n* = 5 per group.

To investigate the hypothesis that suppressive, cross-reactive WT spike antibodies were lowering the Ad-o booster immunogenicity when administered IM, a passive serum transfer experiment was performed (Fig. 6 A). In brief, sera generated from different WT spike vaccines (Novavax NVX-CoV2373, Pfizer-BioNTech BNT162b2, and ChAdOx1) were intravenously (IV) injected into naive recipient mice. Mice were then vaccinated IM with Ad-o, with immune responses assessed a further 3 wk later. Additionally, to assess whether anti-ChAdOx1 (“anti-vector”) antibodies were suppressive to the latter Ad-o response and part of the Ad-WT imprint, we transferred serum into a separate group that was derived from mice primed and boosted with the same vector encoding an irrelevant GFP antigen (ChAdOx1-GFP; “Ad-GFP”).

A range of WT spike–reactive IgG levels were detected between groups prior to IM administration of Ad-o (Fig. 6 B). Mice that received sera from Novavax-WT^IM^+Novavax-WT^IM^–vaccinated mice had the highest levels of transferred anti-WT IgG, followed by those that received Pfizer-WT^IM^+Pfizer-WT^IM^ sera, and then Ad-WT^IM^+Ad-WT^IM^ sera. Only mice that received ChAdOx1 vaccine sera had detectable anti-ChAdOx1 IgG (Fig. 6 B). Anti-WT spike IgG levels following serum transfer of Novavax-WT^IM^+Novavax-WT^IM^- or Pfizer-WT^IM^+Pfizer-WT^IM^–vaccinated mice were equivalent to or higher than serum levels seen in mice directly IM vaccinated with Ad-WT, confirming the level of serum IgG was physiologically relevant to that expected in mice following in vivo responses.

Omicron spike–reactive IgG and o-ACE2^comp^-Abs were measured in recipient mouse serum after transfer to assess the differences in the baseline level of cross-reactivity prior to the vaccination with Ad-o (Fig. 6 C). Levels of omicron spike–reactive IgG were detected in mice that received Ad-WT^IM^+Ad-WT^IM^, Novavax-WT^IM^+Novavax-WT^IM^, and Pfizer-WT^IM^+Pfizer-WT^IM^, and were strongly associated with overall anti-WT spike antibody levels. Low but detectable levels of o-ACE2^comp^-Abs were measured in mice that received Novavax-WT^IM^+Novavax-WT^IM^ and Pfizer-WT^IM^+Pfizer-WT^IM^ sera as well.

3 wk after Ad-o vaccination, mice that received vaccinated mouse sera had reduced omicron spike–specific IgG and o-ACE2^comp^-Ab levels compared with control mice that received naive sera (Fig. 6 D). The omicron spike IgG levels were significantly lower than those following naive serum transfer control group in mice that received Ad-WT^IM^+Ad-WT^IM^ (P ≤ 0.0001), Novavax-WT^IM^+Novavax-WT^IM^ (P = 0.0055), and Ad-GFP^IM^+Ad-GFP^IM^ (P = 0.0014) sera. Likewise, these groups had significantly lower o-ACE2^comp^-Abs than the control naive serum transfer group (Ad-WT^IM^+Ad-WT^IM^, P = 0.0127; Novavax-WT^IM^+Novavax-WT^IM^, P ≤ 0.0001; Ad-GFP^IM^+Ad-GFP^IM^, P = 0.0421). Mice that received Pfizer-WT^IM^+Pfizer-WT^IM^ sera had lower levels of omicron-specific response to the naive control; however, these differences were not statistically significant.

As the transferred sera had some level of omicron-reactive IgG (Fig. 6 C) that could have contributed to the postvaccination responses measured (Fig. 6 D), delta change in omicron-specific response was calculated (omicron after transfer to after Ad-o) to assess the degree of de novo response to Ad-o that was induced by Ad-o vaccination (Fig. 6 E). Differences in omicron-specific IgG titers indicated that mice that received sera from mice vaccinated with a WT spike vaccine had generated much lower de novo omicron spike IgG in response to Ad-o vaccination than the naive group. For mice that received sera from Pfizer-WT^IM^+Pfizer-WT^IM^ or Novavax-WT^IM^+Novavax-WT^IM^ conditions, the median change in omicron-specific IgG titers was negative, demonstrating that de novo omicron-specific responses were not induced by the Ad-o booster vaccine (Fig. 6 E). In contrast, mice receiving sera from Ad-WT^IM^+Ad-WT^IM^–vaccinated animals, which had the lowest pretransfer titer of WT- and omicron-specific IgG, did induce responses with IM Ad-o booster, but these were significantly reduced compared with mice receiving naive sera as a control (Fig. 6 E). Change in o-ACE2^comp^-Ab levels followed the same pattern (Fig. 6 E). There was a strong inverse correlation in the titer of WT-specific IgG present in the transferred serum and the induction of omicron-specific IgG (rho = −0.77) and o-ACE2^comp^-Ab activity (rho = −0.81) by the Ad-o booster vaccine (Fig. 6 F). In contrast, mice that received sera from Ad-GFP^IM^+Ad-GFP^IM^–vaccinated mice had omicron-specific IgG titers that were not significantly reduced compared with controls, and there was a modest, but significant, reduction in o-ACE2^comp^-Ab levels (P < 0.01).

In summary, transferring sera derived from WT spike vaccination prior to Ad-o vaccination resulted in a reduction of the latter omicron-specific responses to Ad-o. Additionally, anti-ChAdOx1 (anti-vector) IgG was shown to suppress the latter Ad-o response, however, to a lesser extent than anti-WT spike IgG. The quantity of anti-WT spike IgG transferred was shown to have a tight inverse correlation with omicron-specific response to Ad-o.

### IM WT vaccine–derived antibodies do not suppress the latter mucosal omicron vaccine boost response

IM omicron vaccine responses were shown to be suppressed by Ad-WT^IM^-derived antibodies that form part of the immunological imprint. Given the mucosal immune compartment is anatomically distinct from the wider systemic compartment, we next assessed whether an IN omicron vaccine response was similarly affected by anti-WT spike antibody responses.

**Figure 7.**
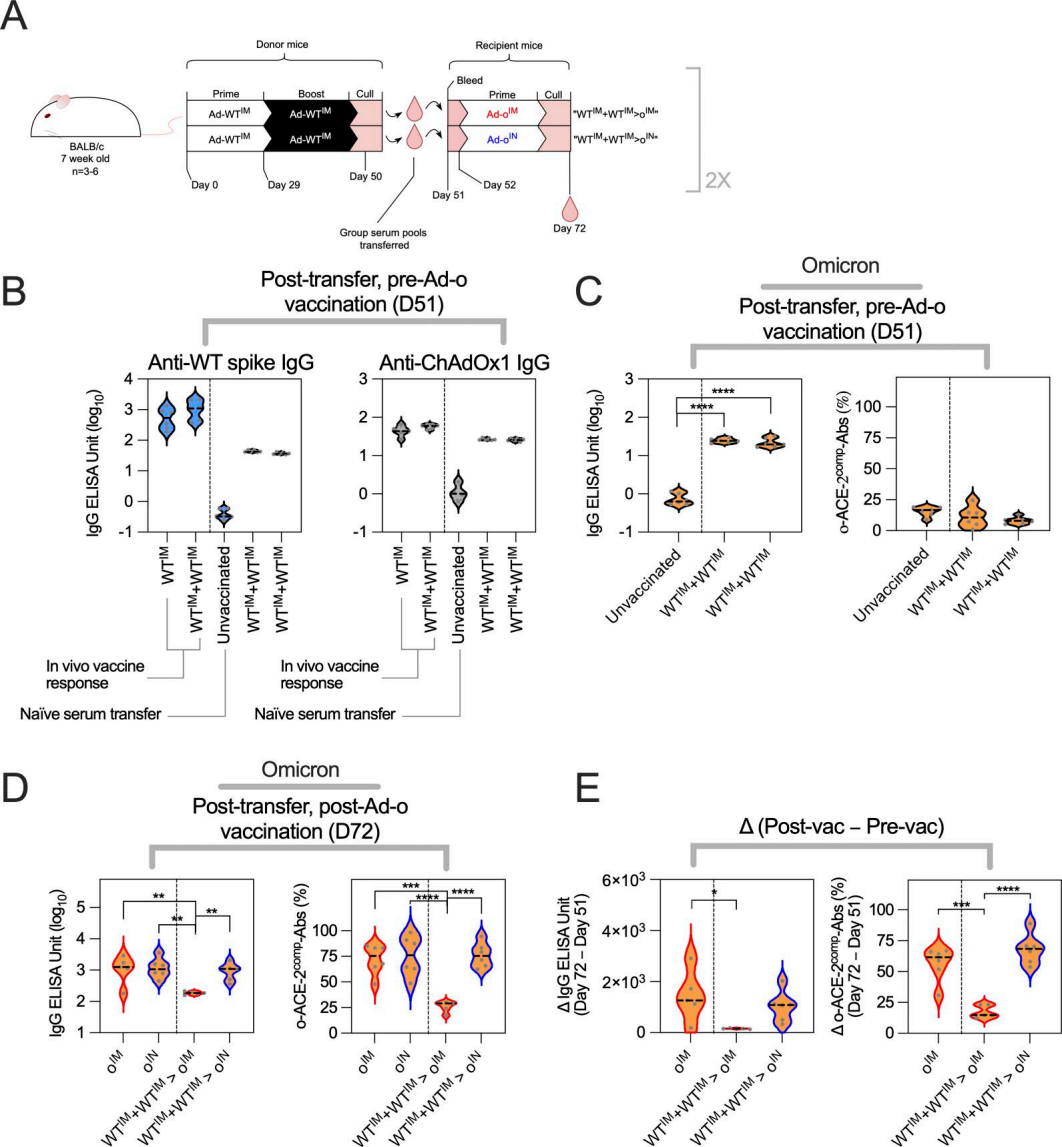
**IM WT vaccine-derived antibodies do not suppress the latter mucosal omicron vaccine boost response. (A)** Vaccination and serum transfer schedule. Donor mice were vaccinated, and then, sera were collected and transferred to naive recipient mice. Recipient mice were vaccinated with Ad-ο IM or IN. Sera from recipient mice were collected after transfer of sera before vaccination (day 51) and 3 wk after Ad-ο vaccination (day 72). **(B)** Levels of anti-WT spike IgG and anti-ChAdOx1 IgG in recipient mouse sera, after transfer of donor sera, and before Ad-ο vaccination (day 51). Reference negative control levels of IgG on day 51 in mice that received naive sera were also included in the figure. **(C)** Levels of anti-omicron spike IgG and o-ACE2^comp^-Abs in mouse sera, after transfer of donor sera, and before Ad-o vaccination (day 51). **(D)** Levels of anti-omicron spike IgG and o-ACE2^comp^-Abs in mouse sera, after transfer of donor sera, and after Ad-o vaccination (day 72). Reference groups following Ad-o^IM^ and Ad-o^IN^ without serum transfer are included. **(E)** Absolute change (day 72–day 51) in anti-omicron spike IgG and o-ACE2^comp^-Ab levels. For data in C–E, a one-way ANOVA test with post hoc comparison between all groups was performed. For all data, *P < 0.05, **P < 0.01, ***P < 0.001, ****P ≤ 0.0001. On violin plots, the dashed black line represents the group median and dots represent individual mice. Data are representative of two independent experiments, where experiment 1 (shown) was *n* = 6 per group and experiment 2 (*n* = 3–6 per group) was *n* = 6 for test group “WT^IM^+WT^IM^>o^IM^” and *n* = 3 for test group “WT^IM^+WT^IM^>o^IN^.”

Akin to the previous antibody transfer experiment, sera from Ad-WT^IM^+Ad-WT^IM^–vaccinated mice were transferred into naive mice. Recipient mice were vaccinated with omicron vaccine either IM or IN (Fig. 7 A). After transfer, prevaccination levels of WT spike IgG and ChAdOx1 were comparable between both groups (Fig. 7 B). Baseline levels of omicron-reactive IgG were detected following Ad-WT^IM^+Ad-WT^IM^ transfer above negative control (naive serum transfer); however, o-ACE2^comp^-Ab levels were very low and comparable to the negative control (Fig. 7 C).

3 wk after IN omicron vaccination, the levels of serum omicron spike–specific IgG and o-ACE2^comp^-Abs were of similar level to those following Ad-o^IN^ and Ad-o^IM^ prime-only regimens that did not receive antibodies. IgG and o-ACE2^comp^-Abs were significantly higher following Ad-o^IN^ than levels following Ad-o^IM^ (IgG: P = 0.0064 and o-ACE2^comp^-Abs: P ≤ 0.0001; Fig. 7 D). A greater absolute change in serum omicron spike–specific IgG and o-ACE2^comp^-Abs following vaccination was also observed in the Ad-o^IN^ group, compared with the Ad-o^IM^ group (IgG: P = 0.0002 and o-ACE2^comp^-Abs: P ≤ 0.0001; Fig. 7 E). Thus, IN Ad-o responses were not suppressed by circulating WT spike vaccine–induced antibodies, whereas IM Ad-o responses were.

### MBCs from IM WT prime minimally participate in latter mucosal omicron vaccine boost responses

**Figure 8.**
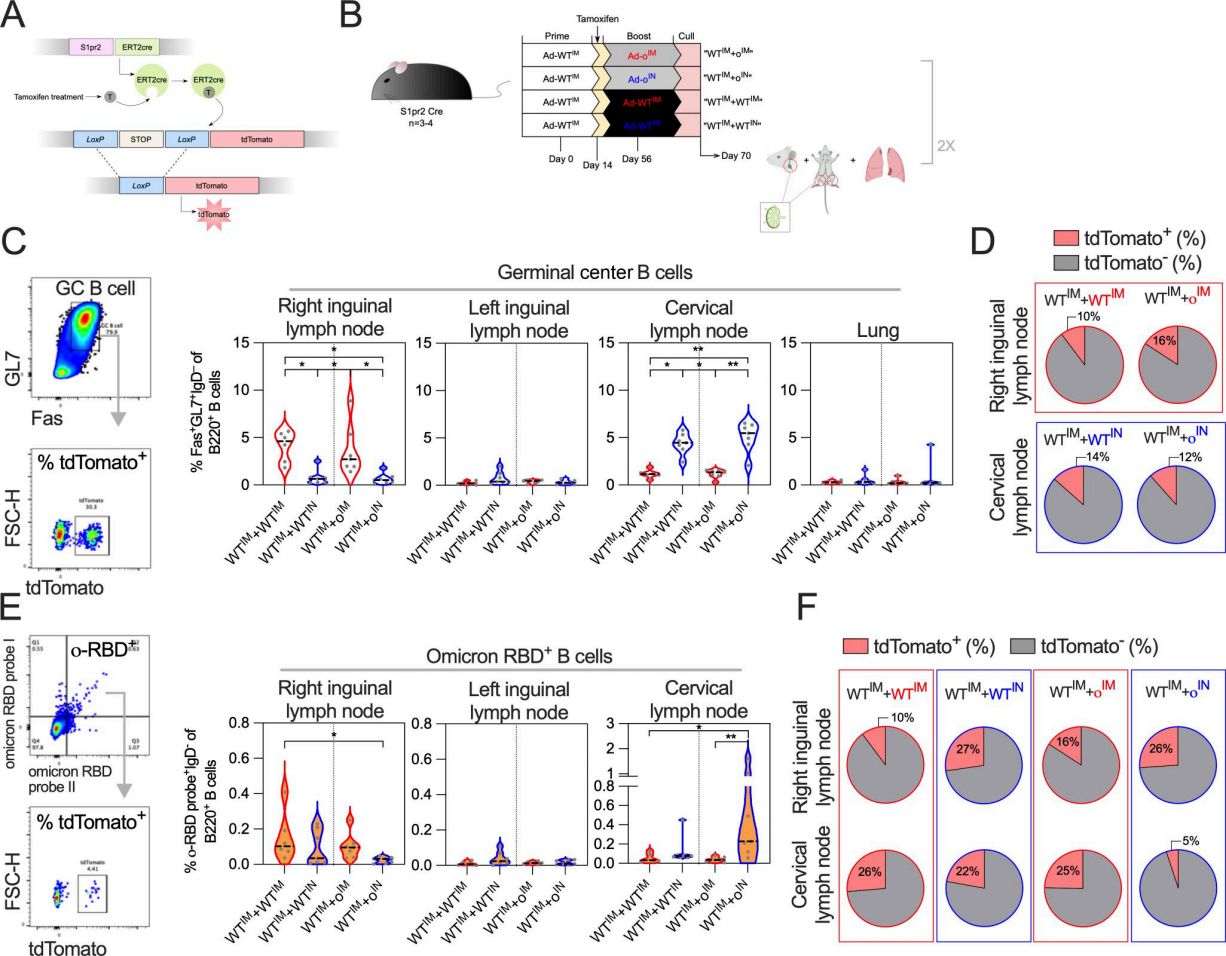
**MBCs from IM WT prime minimally participate in latter mucosal omicron vaccine boost lung response. (A)** Schematic of how transgenic “S1pr2-Cre tdTomato” fate tracking mice tag B cells expressing *S1pr2* and are thus within the GC. **(B)** Vaccination schedule using S1pr2-Cre tdTomato fate tracking mice. Tamoxifen was administered 14 days after prime at time of optimal GC B cell production. Mice were culled 2 wk after boost, with LILN and RILN, respectively, CLN, and lungs collected for analysis. **(C)** Frequencies of GC B cells (Fas^+^GL7^+^IgD^−^B220^+^) in LILN, RILN, CLN, and lungs as measured by cell staining and flow cytometry. Example flow plots for the gating of GC B cells, and the determination of the frequency of tdTomato^+^ GC B cells is located on the left. **(D)** For RILN and CLN cells that had measurable frequencies of GC B cells, the proportion of Ad-WT^IM^ prime-derived (tdTomato^+^) cells (median group response) were indicated in pie charts. **(E)** Frequencies of o-RBD^+^ B cells (IgD^-^B220^+^) in LILN, RILN, and CLN. Example flow plots for the gating of o-RBD^+^ B cells, and the determination of the frequency of tdTomato^+^ GC B cells is located on the left. **(F)** Proportion of o-RBD^+^ B cells that were Ad-WT^IM^ prime-derived (tdTomato^+^) cells (median group response). For data in C and E, a one-way ANOVA test with post hoc comparison between all groups was performed. For all data, *P < 0.05 and **P < 0.01. On violin plots, the dashed black line represents the group median and dots represent individual mice. Data are pooled from two independent experiments (*n* = 3–4 per group per experiment).

**Figure S5.**
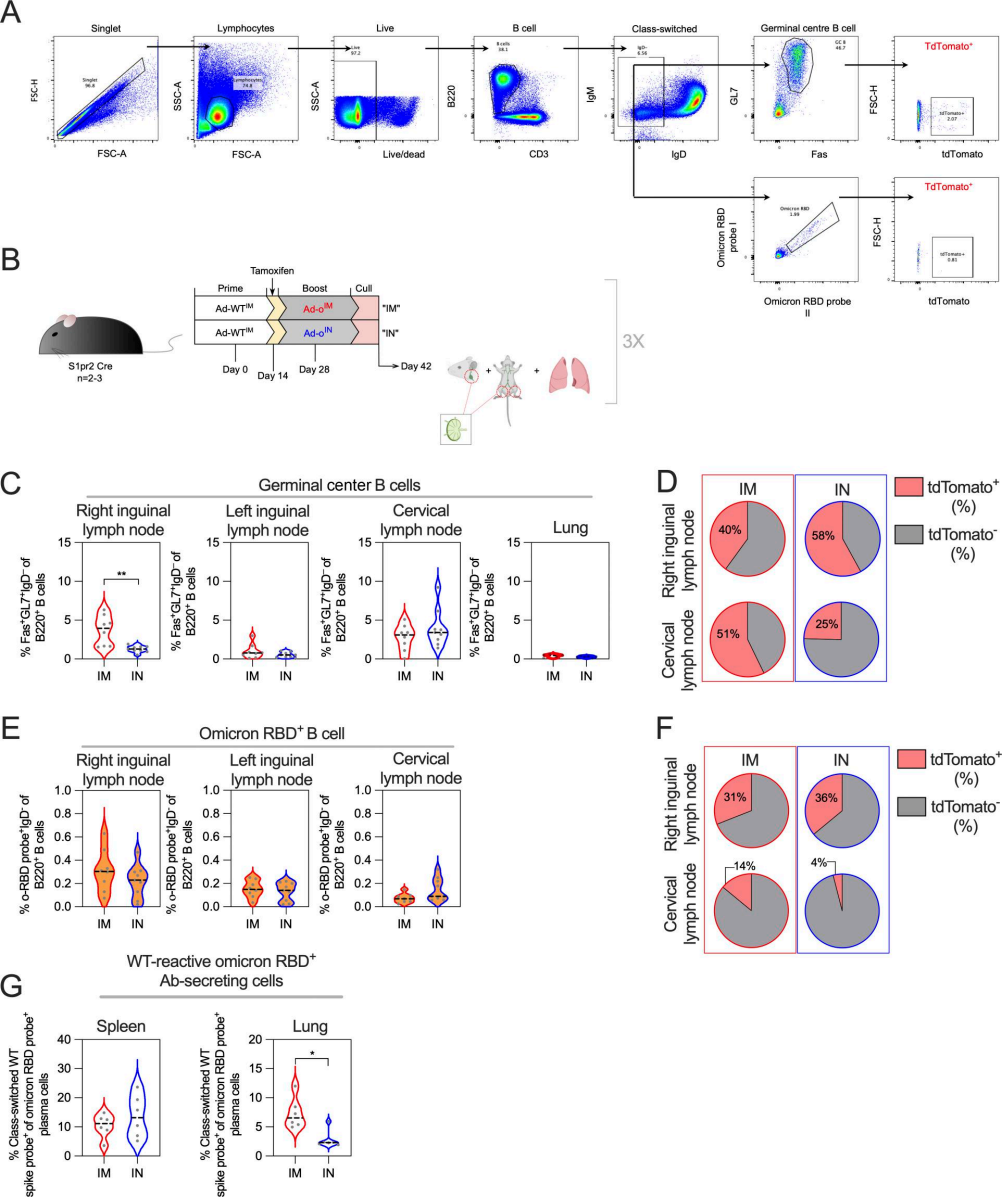
**Minimal participation of MBCs from IM WT prime after 28 days of mucosal boosting. (A)** Gating strategy for experiment featured in Figs. 8 and S5. **(B)** Vaccination schedule using S1pr2-Cre tdTomato fate tracking mice. Tamoxifen was administered in mice 14 days after prime at time of optimal GC B cell production, and mice were boosted on day 28. Mice were culled 2 wk after boost, with LILN and RILN, respectively, CLN, and lungs collected for analysis. The data are compiled from three separate experiments. **(C)** Frequencies of GC B cells (Fas^+^GL7^+^IgD^−^B220^+^) in LILN, RILN, CLN, and lungs as measured by cell staining and flow cytometry. **(D)** For RILN and CLN cells that had measurable frequencies of GC B cells, the proportion of Ad-WT^IM^ prime-derived (tdTomato^+^) cells (median group response) were indicated in pie charts. **(E)** Frequencies of o-RBD^+^ B cells (IgD^−^B220^+^) in LILN, RILN, and CLN. **(F)** Proportion of o-RBD^+^ B cells that were derived from the Ad-WT^IM^ prime (tdTomato^+^) cells (median group response) were indicated in pie charts. **(G)** Frequencies of o-RBD probe–specific spleen and lung PCs that were WT spike probe–reactive (CD19^−^CD138^+^IgD^−^IgM^−^o-RBD^+^WT-S^+^). Data were produced from the experiment featured in Fig. 1. Mann–Whitney tests were completed to test for statistically significant differences between groups (*P < 0.05). For data in C and E, a *t* test was performed. **P < 0.01. On violin plots, the dashed black line represents the group median and dots represent individual mice. Data shown are pooled from three independent experiments (*n* = 2–3 per group per experiment).

As responses following IN administration of omicron vaccine were not suppressed by WT spike antibodies, and IN boosting with omicron vaccine in WT-primed mice resulted in strong omicron-specific responses, we sought to further investigate whether a de novo–type B cell response was occurring locally in the respiratory mucosa, rather than a recall response of the primary cohort of MBCs derived from Ad-WT prime vaccination. To track B cells derived from Ad-WT vaccination, we utilized B cell reporter transgenic mice whereby administration of tamoxifen leads to the permanent expression of tdTomato by germinal center B cells (GC B cells) (i.e., antigen-experienced), as well as their descendants (Shinnakasu et al., 2016) (Fig. 8 A). These transgenic mice were primed IM with Ad-WT (via injection into the right thigh muscle), and then 56 days later either boosted IM (into the same thigh) or IN with Ad-o (Fig. 8 B), with tamoxifen treatment occurring 14 days after prime, a period of optimal GC B cell output, to label prime vaccination GC B cells (Shinnakasu et al., 2016). Mice given a homologous boosting with Ad-WT, either IM or IN, were included as a control. Immune responses in the lungs, cervical lymph nodes (CLN), and right and left inguinal lymph nodes (RILN and LILN) were assessed 2 wk after booster vaccination by cell staining and flow cytometry (Fig. S5 A).

GC B cell responses were strongest in the RILN following IM boosting or the CLN following IN boosting, as expected (Fig. 8 C). GC B cells were defined as cells expressing Fas^+^GL7^+^IgD^−^B220^+^. For such regimens with higher, detectable GC B cells in their RILN (WT^IM^+WT^IM^ and WT^IM^+o^IM^) and CLN (WT^IM^+WT^IN^ and WT^IM^+o^IN^), the proportion of GC B cells that were tdTomato^+^ was minor (median 10–16% of the response) and there were no differences between groups (Fig. 8 D), consistent with other reports of the major role of newly primed B cells in secondary GC responses (de Carvalho et al., 2023; Schiepers et al., 2024). When o-RBD specific B cells were examined (gated on IgD^−^B220^+^ cells, so not limited to GC B cells), again a similar pattern of frequencies across RILN, LILN, and CLN was observed (Fig. 8 E). The o^IN^ boost induced significantly stronger o-RBD–specific B cell responses in the CLN than either boosting IM with either a homologous or heterologous vaccine (P < 0.05 compared with WT^IM^, P < 0.01 compared with o^IM^). A WT^IN^ boost induced intermediate responses, suggestive of some cross-reactivity induced by this regimen. Conversely, the o^IN^ boost had the weakest responses in the RILN (Fig. 8 E).

When tdTomato^+^ cells were examined within the o-RBD–binding population, two patterns were observed. Firstly, o-RBD–binding B cells in nondraining LNs (RILN for IN boost and CLN for IM boost) had a consistent fraction of tdTomato^+^ cells (median 25–27%), suggesting the labeling efficiency of MBCs by a single administration of tamoxifen. Secondly, in the draining LNs (CLN for IN boost and RILN for IM boost), the smallest fraction of tdTomato^+^ cells among the o-RBD–binding fraction within a given draining LN was seen following o^IN^ boost (median 5% versus: 10 % for WT^IM^ boost in RILN, 16% for o^IM^ boost in RILN, and 22% for WT^IN^ boost in CLN; Fig. 8 F).

As these experiments used a 56-day prime-boost interval as opposed to the 28-day interval from earlier experiments, we repeated these experiments with the shortened interval (Fig. S5, B–F). This shorter interval had greater involvement of tdTomato^+^ cells within the overall GC response in the RILN, possibly through GC refueling (Fig. S5 D). However, once again the o^IN^ boost regimen had minimal tdTomato^+^ cells within the o-RBD–binding B cell population in the CLN (median 4%) compared with following an o^IM^ boost (median 31%; Fig. S5 F).

In summary, tdTomato^+^ Ad-WT IM prime–derived B cells comprised a small fraction of the omicron-specific B cell pool in the CLN after heterologous Ad-o IN boost, especially in comparison with the fraction in the RILN pool. The data suggest that the de novo naive B cell population are contributing to the IN Ad-o response following mucosal boosting, in comparison with systemic responses where higher frequencies of Ad-WT IM prime GC B cells have persisted.

In alignment with this finding, we measured a smaller frequency of WT-cross-reactive PCs following Ad-WT^IM^-Ad-o^IN^ vaccination compared with following Ad-WT^IM^+Ad-o^IM^ vaccination within the o-RBD^+^ lung PC population isolated in the initial IN boost experiment (P = 0.0152, Fig. S5 G).

### IM prime-derived, cross-reactive T cells are involved in the latter mucosal boost response in the lungs

IN boosting of Ad-WT^IM^-primed mice with Ad-o not only increased omicron-specific humoral responses otherwise suppressed by Ad-WT^IM^ imprinting, but also induced strong omicron-specific T cell and antibody responses in the respiratory mucosa and broadened the cross-reactive breadth of antibody responses. Since the magnitude of these responses surpassed those elicited from an IN prime of Ad-o alone (Fig. S1), we investigated whether memory T cells derived from Ad-WT^IM^ priming were involved in the latter Ad-o^IN^ response.

**Figure 9.**
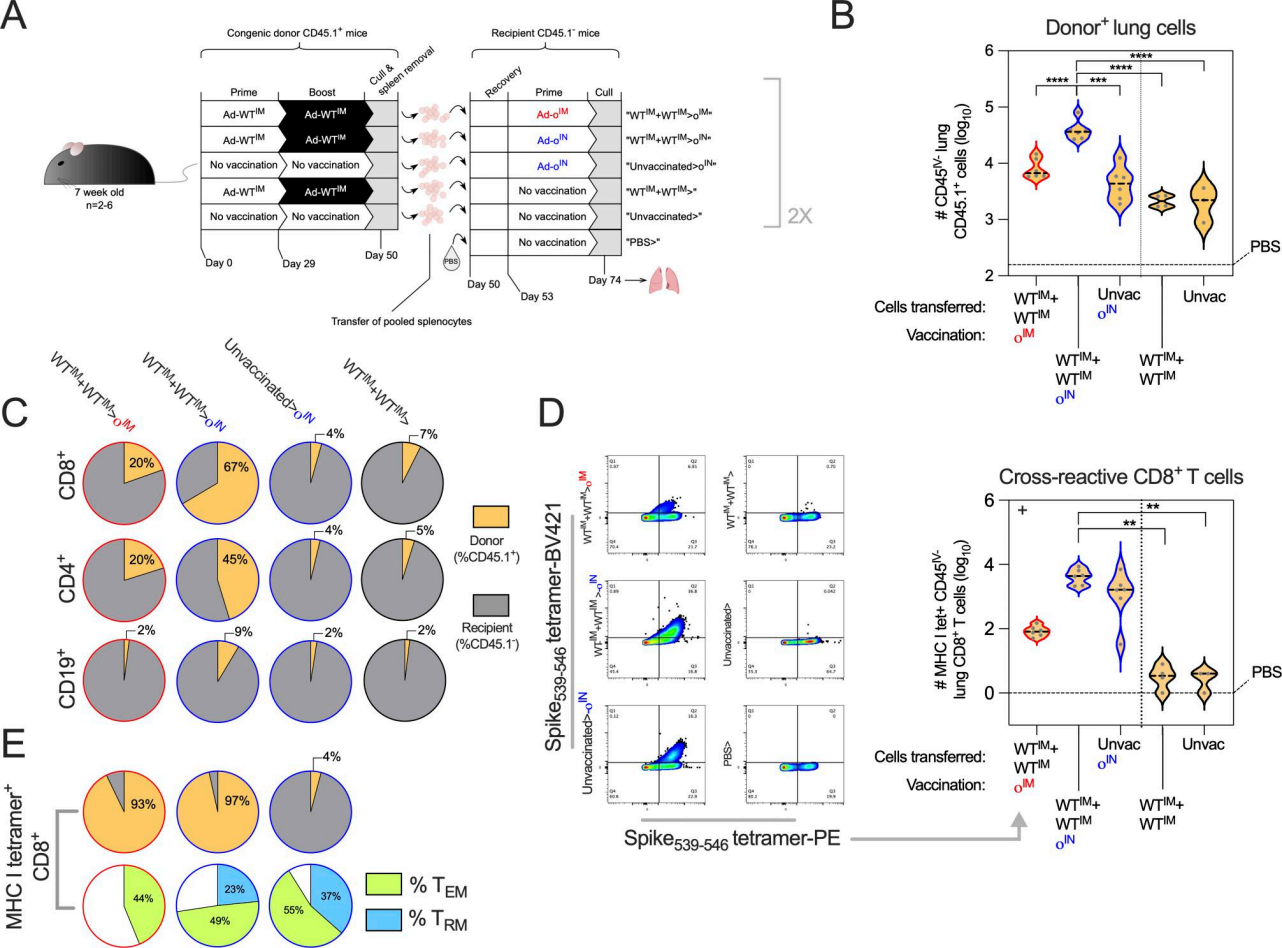
**IM prime-derived, cross-reactive T cells are involved in the latter mucosal boost response in the lungs. (A)** Schedule for the vaccination of donor CD45.1^+^ mice, transfer of donor cells, and subsequent vaccination of recipient CD45.2^+^ (CD45.1^−^) mice. Lungs were harvested 3 wk after Ad-o vaccination. **(B)** Total number (log_10_) of donor^+^ cells (CD45.1^+^CD45^IV−^) in the lungs of mice, as measured via cell staining and flow cytometry. The median response of the negative control mouse group that received PBS instead of cells, and were not vaccinated, is indicated as a dashed horizontal black line. **(C)** Proportion (median for the group) of donor^+^ (CD45.1^+^CD45^IV−^) CD8^+^, CD4^+^, and CD19^+^ cells in the lungs. **(D)** Total number (log_10_) of cross-reactive CD8^+^ T cells (MHC I tet^+^CD45^IV−^) in the lungs of mice binding the conserved H-2K^b^-restricted S_539-546_ epitope, as measured via cell staining and flow cytometry. **(E)** Proportion of donor^+^ cells, and T_RM_^+^ (CD69^+^CD103^+^CD62L^−^CD44^+^) and T_EM_^+^ (CD62L^−^CD44^+^CD127^+^) cells, within the measured cross-reactive lung CD8^+^ T cell (MHC I tet^+^CD45^IV−^) population. For all data, **P < 0.001, ***P < 0.001, ****P ≤ 0.0001. Parametric one-way ANOVA tests were completed when data were normally distributed; otherwise, a nonparametric Kruskal–Wallis test was performed (indicated with a + at the top left of the graph). For multiple comparison testing, the Ad-WT^IM^+Ad-WT^IM^>Ad-o^IN^ regimen was compared against all other regimens. On violin plots, the dashed black line represents the group median response and dots represent individual mice. Data are representative of two independent experiments, where experiment 1 (shown) was *n* = 6 for test groups WT^IM^+WT^IM^>o^IM^, WT^IM^+WT^IM^>o^IN^, and Unvaccinated>o^IN^ and *n* = 4, *n* = 3, and *n* = 2 for groups WT^IM^+WT^IM^>, Unvaccinated>, and PBS>, respectively. In experiment 2 (all *n* = 5), test groups WT^IM^+WT^IM^>o^IM^, WT^IM^+WT^IM^>o^IN^, and critical control group WT^IM^+WT^IM^> were exclusively repeated.

To do this, we devised an adoptive cell transfer experiment using CD45.1^+^ donor mice and CD45.2^+^ (hence CD45.1^−^) recipient mice. To generate WT spike vaccine–derived memory cells, congenic donor mice were either vaccinated Ad-WT^IM^+Ad-WT^IM^ (WT^IM^+WT^IM^) or left unvaccinated, and then, splenocytes harvested from vaccinated mice were then IV transferred into recipient mice (Fig. 9 A). Recipient mice were subsequently vaccinated with either Ad-o IM or IN, or not vaccinated, with immune responses assessed 3 wk after Ad-o vaccination by flow cytometry using anti-CD45.1 antibodies to discriminate between donor- and recipient-derived cells.

The highest number of donor cells in the lungs was observed in mice that received splenocytes from WT^IM^+WT^IM^-vaccinated mice, which were then o^IN^-vaccinated (WT^IM^+WT^IM^>o^IN^) (Fig. 9 B). The number of donor-derived cells (CD45.1^+^) in WT^IM^+WT^IM^>o^IN^ lungs was higher than in mice that received naive (unvaccinated) donor cells prior to Ad-o^IN^ vaccination (WT^IM^+WT^IM^>o^IN^ vs. Unvaccinated>o^IN^; P ≤ 0.0001), suggesting that the WT spike donor cell population had expanded and homed to the lungs upon o^IN^ vaccination. WT^IM^+WT^IM^>o^IN^ CD45.1^+^ lung cells were also significantly higher in number than in mice that received vaccinated-donor cells but remained unvaccinated (vs. WT+WT>No vaccination, P ≤ 0.0001 and vs. Unvaccinated>No vaccination, P ≤ 0.0001).

The frequency of donor-derived (CD45.1^+^) lung CD8^+^ and CD4^+^ T cells, as well as CD19^+^ B cells, was also assessed (Fig. 9 C). A large proportion of lung CD8^+^ T cells in WT^IM^+WT^IM^>o^IN^ mice were donor-derived (median 67% CD45.1^+^) compared with other vaccinated regimens (WT^IM^+WT^IM^>o^IM^, 20% CD45.1^+^; Unvaccinated>o^IN^, 4% CD45.1^+^). Similarly, the largest proportion of donor CD45.1^+^ CD4^+^ T cells were observed following WT^IM^+WT^IM^>o^IN^ regimen (median 45% CD45.1^+^). In contrast, the proportion of donor CD45.1^+^ B cells across all regimens was low, ranging from 2 to 9% CD45.1^+^. The largest contribution of donor CD45.1^+^ B cells was in the WT^IM^+WT^IM^>o^IN^ group, consistent with the T cell responses. Therefore, CD8^+^ and CD4^+^ T cells derived from WT^IM^+WT^IM^ vaccination, but not B cells (consistent with Fig. 8), contributed considerably to the latter o^IN^ response in the lungs.

We hypothesized that the major contribution of memory T cells to the o^IN^ vaccine response was due to cross-reactive responses primed by Ad-WT. The immunodominant H-2K^b^–restricted S_539-546_ epitope is conserved between WT and omicron, and this H-2K^b^ tetramer was used to stain for cross-reactive T cells in the lungs. WT^IM^+WT^IM^>o^IN^ and Unvaccinated>o^IN^ regimens resulted in the generation of more cross-reactive spike-specific CD8^+^ T cells in the lungs than in WT^IM^+WT^IM^>o^IM^ and unvaccinated control groups (WT^IM^+WT^IM^>o^IN^ vs. WT^IM^+WT^IM^>No vaccination, P = 0.0029 and vs. Unvaccinated>No vaccination, P = 0.0076; Fig. 9 D). The proportion of cross-reactive CD8^+^ T cells that were donor-derived was high in groups that received WT^IM^+WT^IM^ cells and were then vaccinated (93% and 97% for WT^IM^+WT^IM^>o^IM^ and WT^IM^+WT^IM^>o^IN^ mice, respectively; Fig. 9 E). In mice that received naive donor cells that were then o^IN^-vaccinated, the majority of cross-reactive CD8^+^ T cells were recipient-derived (96% CD45.1^−^), as expected.

Out of the MHC I tetramer^+^ population following Ad-o vaccination, the T cell phenotype in the lungs was different depending on whether the mice were boosted IN or IM with Ad-o (Fig. 9 E). 44% of the cross-reactive CD8^+^ T cells from WT^IM^+WT^IM^>o^IM^ mice were an effector memory phenotype with no T_RM_ detected (Fig. 9 E). Cross-reactive CD8^+^ T cells from WT^IM^+WT^IM^>o^IN^ mice were 49% T_EM_ phenotype and 23% T_RM_ phenotype. Cross-reactive CD8^+^ T cells from the Naive>o^IN^ group, where the response was recipient-derived, were 55% T_EM_ phenotype and 37% T_RM_ phenotype. Thus, while both Ad-o^IM^ and Ad-o^IN^ drove secondary expansion of cross-reactive memory T cells, Ad-o^IN^ was uniquely superior at recruiting these cells to the lungs and converting them to a T_RM_ phenotype.

Therefore, T cells derived from Ad-WT^IM^ priming contribute substantially to the lung T cell response following a boosting Ad-o^IN^ vaccination.

## Discussion

Updated variant booster vaccines against SARS-CoV-2 are typically administered via the IM route. This route is effective in inducing immunity for protection against severe disease, but less effective at providing sterile protection, with various studies suggesting this is due to the involvement of immunological imprinting restricting the variant IM booster responses, a phenomenon largely described in the context of influenza virus (Collier et al., 2023; King et al., 2023; Tortorici et al., 2024; Wang et al., 2023a; Zaeck et al., 2024). Imprinting derived from sequential WT spike vaccination may limit the protection conferred by variant booster vaccines by suppressing de novo responses to variant spike and perpetuating the ancestral WT spike response. In this study, we utilized alternate routes of vaccine delivery to delineate the mechanisms underpinning immunological imprinting. We demonstrate the profound impact that preexisting immunity can have on sequential immunization humoral responses, while minimal impact is observed in the T cell responses. We demonstrate that by altering route of vaccine administration, the effect of preexisting antibody immunity can be circumvented, and immune responses in the mucosa at the site of infection can be advantageously generated.

When the Ad-o vaccine was administered IM in Ad-WT–primed mice, it was unable to induce substantial omicron-specific humoral responses. The majority of omicron-reactive antibodies produced following the Ad-WT^IM^+Ad-o^IM^ regimen were WT-reactive, suggesting they were derived from a back boosting response to WT spike induced initially with the prime vaccine, rather than de novo response to omicron booster vaccine antigen. Back boosting was also indicated by the higher neutralization of WT pseudotyped virus in sera following Ad-WT^IM^+Ad-o^IM^ vaccination compared with Ad-WT^IM^ prime-only samples. Although Ad-WT^IM^+Ad-o^IM^ vaccination generated omicron-reactive IgG, the neutralization capacity of this IgG was low, and levels of neutralization were lower than that of sera following Ad-o^IM^ prime-only, indicating suppression of a de novo Ad-o^IM^ response. Using passive transfer of serum, we confirmed that WT spike vaccine–derived antibody was mediating this suppressive effect of immunological imprinting. The transferred WT spike–reactive antibodies, which were shown to be cross-reactive with omicron spike, may be inhibiting omicron vaccine immunogenicity in what has been previously referred to as “antigenic blunting”: cross-reactive antibody binding and masking omicron antigen expressed following vaccination, preventing B cell recognition and downstream omicron-specific antibody responses (McNaughton et al., 2022; Zimmermann et al., 2019). In addition to this, these cross-reactive antibodies may be facilitating the accelerated effector cell–mediated clearance of omicron antigen-expressing cells upon vaccination in an Fc-dependent manner, also impairing the ability of B cell to recognize antigen (Dangi et al., 2023). This blunting effect caused by preexisting antibodies could potentially also inhibit boosting against epitopes shared with the antigen used in the priming vaccine. Consistent with this, we observed increased induction of not only omicron-reactive antibodies by IN boost, but also antibodies reactive for ancestral variants.

Importantly, primary addiction is not solely described as an antibody-driven phenomenon; the recall of preexisting WT spike vaccine–derived primary B cell cohorts, which then perpetuate such suppressive antibody responses, is also likely a key element underpinning the imprinting process (Schiepers et al., 2023). The recall of primary B cell cohorts was directly observed in the GC B cell fate tracking experiments, where following IM boosting, a fraction of the response was derived from the primary B cell response, and indirectly observed through the aforementioned back boosting observed in sera. Thus, blunting of IM boosting responses appears to be caused by a combination of serologic and cellular factors that alter targeting and strength of boosting responses.

Other clinical and preclinical studies have observed the impairment of both omicron vaccine responses and omicron breakthrough responses as a result of a preexisting immunological imprint derived from WT spike vaccination (Huang et al., 2023; Tortorici et al., 2024; Wang et al., 2023a; Yisimayi et al., 2024). One clinical study found that triple-WT-vaccinated, omicron-infected individuals mounted recall responses to shared regions of spike between WT and omicron variant, yet failed to mount a de novo response to omicron antigen (Pušnik et al., 2024). In another clinical study, monovalent WT spike–encoding, and bivalent WT and BA.5 spike–encoding mRNA booster vaccines induced comparable antibody responses in individuals; the WT spike antigen within the bivalent vaccine was suggested to have contributed a “deep immunological imprint” limiting the booster vaccine’s potential (Wang et al., 2023a). Our data indicate that even when monovalent omicron vaccine is administered without WT antigen, a preexisting WT imprint is capable of suppressing the omicron vaccine response. Other preclinical studies using different vaccine platforms have noted a similar inefficacy of heterologous IM omicron booster to this present study (Costa Rocha et al., 2024; Cotter et al., 2023; Hawman et al., 2022, *Preprint*).

When the Ad-o omicron vaccine was alternatively administered IN, strong omicron-specific responses were elicited both locally in the respiratory mucosa and systemically. The benefit of an IM-IN or prime-pull regimen has previously been demonstrated with WT spike–based vaccines (Koolaparambil Mukesh et al., 2025; Lapuente et al., 2021; Mao et al., 2022; Shamseldin et al., 2023; Tang et al., 2022). Studies have noted enhanced systemic responses, as well as increased respiratory mucosal IgA, IgG, NAbs, and lung T_RM_ levels, following the IM-IN regimen when compared to regimens solely involving IN or IM vaccination (Koolaparambil Mukesh et al., 2025; Lapuente et al., 2021; Li et al., 2022b; Mao et al., 2022; Shamseldin et al., 2023; Tang et al., 2022). More recently, preclinical studies have tested heterologous IN boosting with a range of different omicron vaccines in WT spike–primed mice, including a recombinant omicron RBD-nucleoprotein fusion protein booster, an MVA booster encoding omicron spike, and an Ad5-based omicron spike booster (Cotter et al., 2023; Lam et al., 2022; Wang et al., 2023b). In alignment with our findings, such studies described enhanced omicron-specific respiratory mucosal immunity, and reduced omicron replication upon challenge, when a booster is administered IN. Although data on heterologous strain mucosal boosters are lacking in humans, two clinical trials using Ad vectors administered as a mucosal boost also found enhanced systemic immunity compared with an intramuscular boost (Li et al., 2022a; Singh et al., 2023), confirming the general benefit of mucosal boosting as seen in mouse models.

In this study, however, we additionally demonstrated that IN but not IM administration of omicron vaccine could bypass the suppressive WT spike vaccine–derived antibody imprinting. This may be explained by the anatomically and immunologically distinct nature of the mucosal immune compartment; circulating antibodies may not have as great a suppressive effect within the mucosal compartment (Holmgren and Czerkinsky, 2005). The lack of suppression by antibodies, in combination with a more “naive” environment, may allow for a more de novo-like B cell response to omicron antigen in the lungs. In alignment with this hypothesis, Schiepers et al. described a suppressive effect of preexisting antibody on MBC recall responses, which prevented MBC participation in latter GC responses (which were subsequently comprised largely of naive B cells) (Schiepers et al., 2024). From the transgenic and adoptive transfer cell tracking experiments, we measured a minimal participation of Ad-WT^IM^–derived MBC on the latter mucosal Ad-o^IN^ response; these data support a more de novo–type B cell response in the lungs and lung-draining LNs. Nevertheless, a very small fraction (9%) of donor-derived B cells (relative to T cell fractions) was measured that had expanded and homed to the lungs following IN boost; these may have also contributed to the broadly reactive antibody pool in the mucosa. In another clinical study, Pušnik et al. showed that unlike cross-reactive T cell responses originating from WT spike vaccination that were stimulated after omicron breakthrough infection, B cell responses to the mutated regions of omicron spike were impaired due to the preexisting vaccine imprint (Pušnik et al., 2024).

Recent studies have elegantly demonstrated that IN administration of an unadjuvanted protein as a boost induced mucosal immunity through the recruitment of MBCs into the lungs and lung-draining LNs (Kwon et al., 2025; Lin et al., 2025). However, a major difference with our study is the use of a boosting antigen that is sequence-matched (i.e., homologous) to the original priming strain. Thus, the lack of MBC recall we observed following Ad-o^IN^ boost and instead the induction of a de novo response is likely due to the sequence divergence compared with the WT priming antigen, consistent with data in humans that omicron booster vaccines induce primarily de novo B cell responses (Alsoussi et al., 2023). Therefore, both the route of delivery and the nature of the boosting antigen have implications for how the immune system responds to a boosting vaccine.

The Ad-WT^IM^+Ad-o^IN^ regimen induced a highly cross-reactive antibody response within the respiratory mucosa and systemically, and superior omicron-specific response when compared with Ad-o administered IN as a single dose. We therefore investigated whether T cells derived from Ad-WT^IM^ prime may be enhancing the Ad-o^IN^ response. We noted that cross-reactive memory CD8^+^ T cells derived from Ad-WT^IM^ vaccination recalled into the lungs upon Ad-o^IN^ vaccination. We also measured the expansion and homing of Ad-WT^IM^ prime–derived CD4^+^ T cells to the lungs. These preexisting T cells likely assist in and contribute to the latter Ad-o^IN^ B cell response, and drive the broad, cross-reactive responses measured locally and systemically. Some of the Ad-WT^IM^–derived memory CD8^+^ T cells converted to a T_RM_ phenotype upon IN boost, as evidenced by the expanded cross-reactive T_RM_ population measured after IN boosting in mice that received Ad-WT^IM^-Ad-WT^IM^ splenocytes. CD8^+^ cells have been shown to migrate from circulation to nonlymphoid tissues upon secondary antigen exposure and adopt a T_RM_-type phenotype (Lucas et al., 2024). The cross-reactivity of T cells derived from ancestral antigen exposure to omicron antigen has been extensively reported, and it is believed this immunity contributes to the blunted disease elicited via omicron in most individuals (Flemming, 2022). We propose that cross-reactive T cells positively contribute to the local lung and systemic responses upon Ad-o^IN^ boost.

One limitation to our conclusions is the use of mouse models that are naive to SARS-CoV-2 infection. We found that an IN prime with Ad-WT induced an imprint that was not bypassed by a IN boost with Ad-o, and instead largely phenocopied the IM prime-boost regimen. These data suggest that upon mucosal exposure, a mucosal imprint is established, analogous to a serologic imprint. Given widespread SARS-CoV-2 infection in the human population, which induces mucosal immunity (Escalera et al., 2024; Mitsi et al., 2023; Puhach et al., 2023), these data may have implications for the use of heterologous boosting vaccines delivered by mucosal exposure. Another limitation is the complication of identifying antibodies induced specifically by the priming versus boosting vaccine, where we were limited to adsorption/depletion experiments. New tools, such as the “K-tag” mouse, will be invaluable to better unravel these questions in future studies (Schiepers et al., 2023).

This study demonstrates that antibodies derived from SARS-CoV-2 WT spike vaccination are suppressive to the latter IM omicron vaccine response, but that alternative mucosal administration of omicron vaccine can increase omicron-specific responses. We show that preexisting memory T cells from IM WT vaccination contribute to the latter omicron IN boost response. It will be critical to establish via human mucosal vaccine delivery trials the efficacy of such vaccines against SARS-CoV-2 variants. Furthermore, how these findings can be extended to other major respiratory pathogens (e.g., RSV or influenza virus) and how they can be incorporated into rationally designed heterologous prime-boost strategies for immunologically naive individuals require careful consideration.

## Materials and methods

### Construction of omicron spike adenovirus vaccine

Ad-o (ChAdOx1-o) was constructed in similar fashion to ChAdOx1 nCoV-19, which was abbreviated as Ad-WT in this paper for simplicity, and formerly marketed as Vaxzevria (INN: ChAdOx1-S) (van Doremalen et al., 2020). In brief, the spike sequence of omicron BA.1 was codon-optimized for expression in human cells, and modified to include a tissue-type plasminogen activator leader sequence. It then was synthesized by GeneArt (Thermo Fisher Scientific). The sequence was then cloned directly into a derivative of bacterial artificial chromosome (BAC) containing the ChAdOx1 genome, as previously described by Dicks et al. (2012); the omicron spike ORF was cloned downstream of a long CMV promoter and upstream of a BGH polyA termination sequence. The ChAdOx1-o genome was then excised from the BAC, and the virus was rescued using T-REx cells (Cat: R71007 [Invitrogen]). The virus was purified by CsCl gradient ultracentrifugation (Cottingham et al., 2012).

### Animal ethics statement and husbandry

Mice were used in accordance with the UK Animals (Scientific Procedures) Act (ASPA) under project license numbers P9804B4F1, PP2352929, PP3430109, and PP2421538, granted by the UK Home Office following ethical review by the University of Oxford Animal Welfare and Ethical Review Board. Animals were group-housed in individually ventilated cages (IVCs) under specific pathogen–free conditions, with constant temperature and humidity with lighting on a 12:12 (8 am to 8 pm) or 13:11 (7 am to 8 pm) light–dark cycle. All animals were humanely sacrificed at the end of each experiment by cervical dislocation. All procedures conducted were followed according to UK Home Office/ASPA regulations by a personal license (PIL)-holder. For hamster infection experiments, studies were approved by the Rocky Mountain Laboratories Institutional Animal Care and Use Committee following the guidelines put forth in the Guide for the Care and Use of Laboratory Animals, eighth edition, the Animal Welfare Act, United States Department of Agriculture, and the United States Public Health Service Policy on the Humane Care and Use of Laboratory Animals. Studies were conducted in an AAALAC international-accredited facility. The Institutional Biosafety Committee (IBC) approved studies with SARS-CoV-2 virus strains under BSL3 conditions. Virus inactivation of all samples was performed according to IBC-approved standard operating procedures for the removal of specimens from high-containment areas.

### Mouse study design and vaccinations

Female BALB/cOlaHsd (Inotiv), C57BL/6JOlaHsd (Inotiv), Ly5.1 congenic C57BL/6J mice (B6.SJL-PtprcaPepcb/BoyCrl; supplied by the Biomedical Sciences Building, Oxford University), and transgenic C57BL/6J S1pr2-ERT2cre Ai9 mice (Shinnakasu et al., 2016; kindly provided by Oliver Bannard, University of Oxford, Oxford, UK) were used for experiments (all 6–7 wk old, female and male, *n* = 2–6 per experiment) (Shinnakasu et al., 2016). Vaccines were administered via IM injection or IN administration, while the mice were anaesthetized. Unless otherwise specified in the figure legend, a 4-wk prime-boost interval was used to align with the standard dosing regimen for mice used in preclinical development of the ChAdOx1 nCoV-19 vaccine (Graham et al., 2020). Short-term anesthesia was achieved using vaporized IsoFlo. Each dose of vaccine was either 10^8^ IU if ChAdOx1 vector (standard dose for preclinical development [Graham et al., 2020]), 5 μg Novavax NVX-CoV2373, or 1 μg Pfizer-BioNTech BNT162b2. For IM vaccinations, a total volume of 50 μl of vaccine (diluted in PBS) was injected into the posterior thigh muscle, with the exception of Novavax vaccine; mice vaccinated with Novavax NVX-CoV2373 received one 50 μl injection in both the right and left posterior thigh muscle. Prime and boost IM injections were completed in the same thigh; for BALB/c and Ly5.1 mouse experiments, this was the left posterior thigh, and for C57BL/6J *S1pr2*-ERT2cre-tdTomato mice, this was the right posterior thigh. IN vaccination involved suspending a total volume of 25–30 μl of vaccine drop-by-drop over the nostrils of the mouse with a pipette, such that the mice were actively inhaling the vaccine.

### Blood, lung, and spleen processing

Blood was either collected via peripheral tail vein bleed with a Microvette capillary tube if sampled after prime vaccination, or via terminal cardiac puncture with syringe if collected at the end of the vaccination schedule. Blood samples were left to clot for a minimum of 30 min, and then centrifuged for separation and collection of the serum fraction. For flow cytometry and ELISpots, lungs and spleens were processed to a single-cell suspension, and reconstituted in complete Minimum Essential Medium, α modification (α-MEM), containing 10% fetal calf serum (FCS), 1% penicillin/streptomycin, and 1% L-glutamine. Lung tissue was minced with scissors and preincubated with a digestion mixture containing collagenase type XI (C7657 [Merck Life Science Ltd]) and DNase type I (D5025 [Merck Life Sciences Ltd]) in α-MEM (1% penicillin/streptomycin and 1% L-glutamine) for 1 h at 37°C, prior to processing. Processing was completed mechanically by pressing tissue through 70-μM cell strainers, and mixture was then treated with ammonium chloride potassium lysis buffer to lyse all erythrocytes. Cells were then counted via a CASY cell counter.

### NALT removal and processing

NALT was collected and processed as previously described (Bissett et al., 2024). In brief, the mouse upper hard palate was removed with a scalpel and cleaned of blood and debris. It was then added to 250 μl RPMI (containing 10% FCS, 1% penicillin/streptomycin, and 1% L-glutamine) in the well of a 48-well cell culture plate (Costar). It was incubated for 3 days at 37°C in a cell culture incubator (5% CO_2_). The supernatant was then added to collection tubes and spun down at 460 g for 5 min to pellet cellular debris. Supernatant was added to collection tubes and stored at −20°C until it was used in assays. A more detailed protocol is described by Cisney et al. (2012).

### BAL fluid collection

To collect BAL fluid, a catheter was inserted into the trachea, and the lungs were then flushed with PBS using a syringe: once with 300 μl of PBS if only collecting antibodies, or an additional time with 1 ml of PBS if collecting BAL cells too. The fluids from each wash were then transferred to separate 1.5-ml collection tubes. The collection tubes were centrifuged at 460 *g* for 5 min to pellet cellular contents. The supernatants were removed and treated with protease inhibitor (1:100 dilution), and stored at −20°C until use in antibody assays If BAL cells were to be analysed, the cellular pellets from both washes per sample were then resuspended in 100 μL PBS and pooled prior to cell staining.

### Cell staining and flow cytometry

To stain cells, cells were first incubated with a mixture of LIVE/DEAD Fixable Near-IR Dead Cell Stain Kit (1:2,000; Cat: L34975 [Invitrogen]), for the experiment in Figs. 1, 2, and 9, or Zombie Yellow Fixable Viability Kit (1:2,000; Cat: 423103 [BioLegend]) for the experiment in Fig. 8, and anti-mouse CD16/CD32 Fc block (1:25, Clone: 2.4G2, Cat: 553141 [BD Biosciences]) in a 96-well round-bottom plate for 30 min in the dark at 4°C. Cells were then washed with PBS with 0.5% bovine serum albumin (BSA). Then, an antibody cocktail was added and plate incubated for 30 min in the dark at 4°C. The antibody cocktail for experiments in Figs. 1 and 2 contained BV650 anti-mouse IgD (1:100; Clone: 11-26c.2a, Cat: 405721 [BioLegend]), AF700 anti-mouse CD62L (1:100; Clone: MEL-14, Cat: 104426 [BioLegend]), PE/Cy7 anti-mouse IgM (1:100; Clone: RMM-1, Cat: 406514 [BioLegend]), PE/Cy5 anti-mouse CD44 (1:100; Clone: IM7, Cat: 103010 [BioLegend]), AF594 anti-mouse CD80 (1:100; Clone: 16-10A1, Cat: 104754 [BioLegend]), BV785 anti-mouse CD138 (1:100; Clone: 281-2, Cat: 142534 [BioLegend]), BV711 anti-mouse CD69 (1:100; Clone: H1.2F3, Cat: 104537 [BioLegend]), Pacific Blue anti-mouse CD19 (1:100; Clone: 6D5, Cat: 115523 [BioLegend]), BV421 anti-mouse CD103 (1:150; Clone: 2E7, Cat: 121421 [BioLegend]), BUV737 anti-mouse CD127 (1:100; Clone: SB/199, Cat: 612841 [BD Biosciences]), Spark Blue 550 anti-mouse CD4 (1:100; Clone: GK1.5, Cat: 100474 [BioLegend]), BUV395 anti-mouse CD273 (1:100; Clone: TY25, Cat: 565102 [BD Biosciences]), PerCP-Cy5.5 anti-mouse CD3 (1:100; Clone: BB23-8E6-8C8, Cat: 561478 [BD Biosciences]), BV570 anti-mouse CD8 (1:100; Clone: 53-6.7, Cat: 100739 [BioLegend]), and BV605 anti-mouse CD73 (1:100; Clone: TY/11.8, Cat: 127215 [BioLegend]). The antibody cocktail for the adoptive transfer experiment in Fig. 9 contained AF350 anti-mouse CD62L (1:100; Clone: 95218, Cat: FAB5761U-100UG [Bio-Techne]), BV510 anti-mouse IgM (1:100; Clone: RMM-1, Cat: 406531 [BioLegend]), BUV563 anti-mouse CD19 (1:100; Clone: 1D3, Cat: 749028 [BD Biosciences]), BUV615 anti-mouse CD45.1 (1:100; Clone: A20, Cat: 751467 [BD Biosciences]), PerCP anti-mouse/human CD44 (1:100; Clone: IM7, Cat: 103036 [BioLegend]), BUV496 anti-mouse CD4 (1:100; Clone: GK1.5, Cat: 612952 [BD Biosciences]), BV650 anti-mouse IgD (1:100; Clone: 11-26c.2a, Cat: 405721 [BioLegend]), PE/Cy7 anti-mouse CD185 (1:50; Clone: L138D7, Cat: 145516 [BioLegend]), APC-eFluor 780 anti-mouse CD8 (1:100; Clone: 53-6.7, Cat: 47-0081-82 [eBioscience]), PE/Dazzle 594 anti-mouse CD103 (1:100; Clone: 2E7, Cat: 121430 [BioLegend]), PE/Cy5 anti-mouse CD69 (1:100; Clone: H1.2F3, Cat: 104510 [BioLegend]), BV785 anti-mouse CD138 (1:50; Clone: 281-2, Cat: 142534 [BioLegend]), BUV737 anti-mouse CD127 (1:100; Clone: SB/199, Cat: 612841 [BD Biosciences]), BUV395 anti-mouse CD273 (1:100; Clone: TY25, Cat: 565102 [BD Biosciences]), and MHC I tetramers (H-2K^b^ SARS-CoV-2 S_539-546_ (VNFNFNGL)) conjugated to PE and BV421 (1:500; [NIH Tetramer Core Facility]). Additionally, 2.5 μg (in 100 μl PBS) of BV510 anti-mouse CD45.2 (Clone: 104, Cat: 109838 [BioLegend], for the experiments in Figs. 1 and 2) or AF700 anti-mouse CD45 (Clone: 30-F11, Cat: 103128 [BioLegend], for the experiment in Fig. 9) was injected into the peripheral tail vein of mice 5 min prior to cull to stain all circulatory leukocytes. For the experiment in Fig. 8, the following surface staining cocktail was used: FITC anti-mouse GL7 (1:100; Clone: GL7, Cat: 144604 [BioLegend]), anti-mouse Fas PerCP-eFluor 710 (1:50; Clone: 15A7, Cat: 46–0951-82 [Invitrogen]), PE/Cy7 anti-mouse CD138 (1:100; Clone: 281-2, Cat: 142514 [BioLegend]), APC-Cy7 anti-mouse NK-1.1 (1:100; Clone: PK136, Cat: 108724 [BioLegend]), APC-Cy7 anti-mouse F4/80 (1:100; Clone: BM8, Cat: 123118 [BioLegend]), BV605 anti-mouse IgM (1:100; Clone: RMM-1, Cat: 406523 [BioLegend]), BV650 anti-mouse CD3 (1:50; Clone: 17A2, Cat: 100229 [BioLegend]), and BV711 anti-mouse IgD (1:100; Clone: 11-26c.2a, Cat: 405731 [BioLegend]). B cell probes were prepared prior to staining and also added to the surface cocktail for the experiment in Figs. 1 and 8; SARS-CoV-2 B.1.1.529 (Omicron) spike RBD protein (His & AVI Tag), Biotinylated (Cat: 40592-V49H7-B-SIB [Sino Biological]) was incubated with Streptavidin, AF647 conjugate (Cat: S21374 [Life Technologies Ltd]) or Streptavidin, APC conjugate (Cat: 405243 [BioLegend]) or Streptavidin, BV421 conjugate (405225 [BioLegend]), in a 4:1 M ratio for 30 min on ice in the dark. This was repeated with biotinylated WT spike (The Native Antigen Company) and PE Streptavidin (Cat: 405203 [BioLegend]), as well as for the experiment in Fig. 1 to stain for cross-reactive B cells. Following incubation, cells were washed and resuspended in 100 μl PBS with 0.5% BSA. Cells were acquired on SONY ID7000 Spectral Cell Analyzer (experiments presented in Figs. 1, 2, and 9) or BD LSR II Flow Cytometer (experiment presented in Fig. 8). Samples were run until all cells were acquired. The gating strategy is outlined in Fig. S2 and Fig. S5 A.

### Spleen IFNγ ELISpots

PVDF-membrane ELISpot plates (Millipore) were coated with 50 μl (per well) of 5 μg/ml anti-mouse IFNγ (Clone: AN18, Cat: 3321-2 [Mabtech]) overnight at 4°C. Plates were then washed with PBS and then blocked by adding 100 μl complete α-MEM media and incubating for 1 h at room temperature (RT). Splenocytes and lung cells processed to single-cell suspension and adjusted to a cell density of 10^7^ cells/ml were added to plates. Each sample was plated in duplicate, and titrated to achieve three cell concentrations: 5E+05, 2.5E+5, and 1.25E+5 (and plated once additionally at 5E+05 for unstimulated control). Then, a peptide pool containing peptides of S1 that contained omicron BA.1-specific amino acid mutations was added for stimulation of the cells over 18 h at 37°C (Table S1). IFNγ spots were detected with biotinylated anti-mouse IFNγ antibody (mAb R4-6A2, Cat: 3321-2 A, biotin [Mabtech], diluted to 1 μg/ml), followed by streptavidin-ALP (Cat: 3321-2 A [Mabtech], diluted to 1 μg/ml). To develop spots, alkaline phosphatase (AP) conjugate substrate (Cat: 1706432 [Bio-Rad]) was added. Spots were counted on an AID ELISpot reader, and data are represented as spot-forming units per million splenocytes.

### Standardized indirect antigen-specific isotype ELISAs

96-well Nunc MaxiSorp plates were separately coated with 50 μl/well of 2 μg/ml (for tIgG detection) or 5 μg/ml (for IgA detection) recombinant SARS-CoV-2 spike variants alpha (B.1.1.7), beta (B.1.351), gamma (P.1), epsilon (B.1.429), delta (B.1.617.2), original spike with a D614G mutation (B.1) (all obtained from AstraZeneca), or omicron spike (B.1.1.529; Cat: REC32008 [The Native Antigen Company]) overnight at 4°C. Plates were then washed with PBS/Tween (0.05% vol/vol), and then blocked for 1 h at RT with Blocker Casein in PBS (Thermo Fisher Scientific). Blocker was then discarded, and casein-diluted samples were plated with a positive standard curve (serially diluted 1:2), negative control samples, and blank (PBS), all in duplicate. For ancestral variant ELISAs (alpha, beta, gamma, delta, WT), a cross-reactive pool of sera was used for the standard curve, such that the curve performs similarly on each antigen; the coefficient of variances of four points within the linear portion of the cross-reactive pool curve were calculated to assess the curve’s performance. For omicron antigen ELISAs, a separate pool of sera from omicron-vaccine–vaccinated mice was used. Samples were incubated for 2 h at RT, with shaking. Plates were washed after sample incubation, and casein-diluted secondary antibody was added to plates for incubation for 1 h at RT; antibodies used were AP-conjugated goat anti-mouse IgG (diluted 1:5,000; Cat: A3562 [Sigma-Aldrich]) and goat anti-mouse IgA-AP (diluted 1:1500; Cat: 1040-04 [Southern Biotech]). Plates were then washed, and developer was added; developer was made by dissolving p-Nitrophenyl Phosphate, Disodium Salt substrate (Sigma-Aldrich) in diluted Pierce diethanolamine substrate buffer (Thermo Fisher Scientific). For IgG and IgA, OD_405nm_ values were interpolated off of the linear portion of the standard curve that was assigned arbitrary ELISA units, and transformed to account for their specific dilutions.

### Cells and virus for infection studies

SARS-CoV-2 variant B.1.1.529 BA.1 (hCoV-19/USA/GA-EHC-2811C/2021, EPI_ISL_7171744) was obtained from Mehul Suthar, Emory University, Atlanta, Georgia. The virus stock was sequenced, analyzed using Bowtie2 version 2.2.9, and no SNPs compared with the patient sample sequence were detected. Virus propagation was performed in Vero E6 cells grown in DMEM (Gibco) supplemented with 2% fetal bovine serum (Gibco), 1 mM L-glutamine (Gibco), 50  U/ml penicillin (Gibco), and 50 μg/ml streptomycin (Gibco) (DMEM2). VeroE6 cells were maintained in DMEM supplemented with 10% fetal bovine serum, 1 mM L-glutamine, 50  U/ml penicillin, and 50 μg/ml streptomycin. *Mycoplasma* testing was performed at regular intervals and was not detected in cells or virus stocks.

### Hamster challenge vaccine dosage optimization

Six (three male, three female) 4- to 6-wk-old Syrian hamsters (Envigo) were anesthetized with isoflurane to effect and vaccinated with Ad-o delivered IM in two 100 μl doses into the posterior thighs 10 and 6 wk prior to challenge. Vaccine dose delivered varied between 10^8^ and 10^2^ infectious units per animal. As a control, nine 4- to 6-wk-old Syrian hamsters were vaccinated with ChAdOx1-GFP using the same regimen. All animals were anesthetized with isoflurane to effect and were challenged using a combined IN (40 μl) and intratracheal (IT, 100 μl) approach with a total of 10^4^ TCID_50_ B.1.1.529 BA.1/animal in sterile DMEM. Body weights were recorded daily. Oropharyngeal swabs were collected daily in 1 ml of DMEM. On day 5, all animals were euthanized, and lung samples were collected for quantitative RT-PCR (qRT-PCR) analysis.

### Hamster SARS-CoV-2 omicron challenge

14 (seven male, seven female) 4- to 6-wk-old Syrian hamsters were randomly allocated per group. Animals were anesthetized with isoflurane to effect and vaccinated with 10^5^ infectious units of Ad-WT (three groups) or ChAdOx1-GFP (one group) via IM injection. The vaccines were administered in two 100 μl doses into the posterior thighs, 10 wk prior to challenge. 4 wk after vaccination, animals were anesthetized with isoflurane to effect and boosted with either 10^5^ infectious units of Ad-WT (IM, as previously described), Ad-o (IM, as previously described), Ad-o (IN, delivered in 40 μl), or ChAdOx1-GFP (IM, as previously described). All animals were anesthetized with isoflurane to effect and challenged using a combined IN (40 μl) and intratracheal (IT, 100 μl) approach with a total of 10^4^ TCID_50_ B.1.1.529 BA.1/animal in sterile DMEM. Body weights were recorded daily. Oropharyngeal swabs were collected daily in 1 ml of DMEM. On days 2 and 5, seven animals per group were euthanized, and serum, lung, and nasal turbinate samples were collected for analysis.

### RNA extraction and qRT-PCR reaction

Swab samples were vortexed, and RNA was extracted using the QIAamp Viral RNA kit (Qiagen). Tissue samples were weighed, homogenized, and extracted using the RNeasy kit (Qiagen) according to the manufacturer’s instructions and following high-containment laboratory protocols. 5 μl of extracted RNA was tested with the QuantStudio 3 system (Thermo Fisher Scientific) according to the manufacturer’s instructions using viral sgRNA-specific assays (Rothe et al., 2020). Ct values were compared with standards containing a known number of genome copies.

### Binding and neutralizing antibody titers against different spike proteins on the MESO QuickPlex

The V-PLEX SARS-CoV-2 Key Variant Spike Panel 1 kit (K15651U; MSD) was used to analyze hamster serum samples on the Meso QuickPlex instrument (K15203D; MSD). For binding IgG titers, a 96-well plate was first incubated with 150 μl of Blocker A solution at RT with shaking for 30 min, followed by three washes with 150 μl/well of MSD Wash Buffer. Standard curve solutions and hamster serum samples, diluted 10,000-fold, were prepared, and 50 μl of each was added to the plate in duplicates. The plate was sealed and incubated with shaking at RT for 2 h, then washed three times with 1× MSD Wash Buffer. An in-house secondary antibody (MSD GOLD SULFO-TAG NHS-Ester [R31AA-2 MSD] conjugated to goat anti-hamster IgG [SA5-10284 Thermo Fisher Scientific]) was diluted 10,000-fold in Diluent 100. A 50 μl volume of this solution was added to each well, and the plate was sealed and incubated with shaking at RT for 1 h. Following incubation, the plate was washed as before, and 150 μl of MSD Gold Read Buffer B was added to each well. The plate was immediately read using the MSD instrument. Antibody concentrations (AU/ml) were calculated using MSD Workbench 4.0 software. For ACE-2 competition titers, manufacturer’s instructions were followed. Percent competition was then calculated using values obtained from serum obtained from animals vaccinated with ChAdOx1-GFP.

### Inguinal and CLN removal and processing

Inguinal and CLN were collected and added to RPMI media containing 5% FCS and 1% penicillin/streptomycin. Tissues were mechanically dissociated through 70-μM cell strainers to obtain single-cell suspensions.

### Mouse serum antibody and adoptive cell transfer experiments

For the serum transfer experiments, sera from vaccinated BALB/cOlaHsd mice were collected and pooled in groups under sterile conditions. A total volume of 100 μl of serum was injected into the peripheral tail vein of each recipient BALB/cOlaHsd mouse. For the adoptive cell transfer experiment, the spleens of vaccinated Ly5.1/CD45.1^+^ congenic mice (B6.SJL-PtprcaPepcb/BoyCrl) were harvested and processed under sterile conditions to single-cell suspension as detailed previously. Splenocytes were pooled in groups, and cell concentrations were determined via Countess 3 Automated Cell Counter. Group cell concentrations were normalized via dilution in PBS. An equivalent of one processed donor spleen in 100 μl total volume (PBS suspension) was transferred via peripheral tail injection into each recipient mouse. For all peripheral tail vein injections, mice were warmed at 37°C for 10 min to optimally dilate the tail veins prior to injection.

### Cell fate mapping experiment

Cre expression in C57BL/6J *S1pr2*-ERT2Cre-tdTomato mice was induced by administering a single dose of 12.5 mg tamoxifen (Sigma-Aldrich) dissolved in corn oil at 50 mg/ml by oral gavage 14 days after prime.

### WT spike IgG depletion ELISA

To measure o-RBD-specific antibodies that are non-WT spike cross-reactive, a protocol based off of that detailed by Pušnik et al. (2024) was used. In brief, the quantity of anti-o-RBD IgG in samples was first determined via indirect standardized ELISA as described above; plates were coated with 2 μg/ml SARS-CoV-2 B.1.1.529 (Omicron) spike RBD (Cat: 40592-V08H121 [Sino Biological]). The subsequent (calculated) EU values were used to normalize each sample to the equivalent quantity of anti-o-RBD IgG, by diluting each sample with casein. Each sample (in duplicate) was then incubated for 1 h at 37°C (shaking) in a flat-bottom 96-well nonabsorbent plate with a range of different concentrations of WT spike (total volume of 100 μl/well); WT spike was titrated from 6 μg/ml to 0.008 μg/ml via 1:3 serial dilutions. A WT spike–free control was also included. After incubation, 50 ml of the mixture was transferred to a pre-o-RBD–coated (2 μg/ml), pre–casein-blocked plate. A standard indirect IgG ELISA was then conducted as described above. Development of each sample curve was stopped when the WT spike–free control reached an OD_405nm_ of 1.00. Curves were plotted and area under the curve values calculated.

### Luminex variant neutralization/ACE-2 competition assay

To assess the capacity of antibody sera to inhibit SARS-CoV-2 variants alpha (B.1.1.7), beta (B.1.351), gamma (P.1), delta (B.1.617.2), omicron (B.1.1.529), and WT from binding to ACE-2, the Neutralizing Antibody 6-Plex ProcartaPlex Panel kit (Thermo Fisher scientific) was used, with user manual instructions followed. In brief, serum samples were diluted 1:400 in assay buffer and incubated with washed Luminex beads coated with either recombinant alpha (B.1.1.7), beta (B.1.351), gamma (P.1), delta (B.1.617.2), WT or omicron (B.1.1.529) spike S1 (except for gamma, which was full spike) on a shaker at RT for 2 h. After washing, biotinylated recombinant ACE-2 was added and plate incubated on a shaker at RT for 30 min. Beads were then washed, and incubated for an additional 30 min at RT (shaking) with streptavidin-PE. After a final wash, beads were acquired in reading buffer on a MAGPIX Luminex machine. ACE-2 competition was represented as a percentage, with 0% inhibition defined as the mean fluorescence intensity of the internal plate blank; the blank sample will enable complete binding of spike to ACE-2 and generate the upper MFI limit.

### WT and omicron lentivirus pseudoneutralization assay

To assess the capacity of sera to neutralize SARS-CoV-2 variants WT and omicron (B.1.1.529) virus via blocking of lentiviral entry into target cells, omicron- and WT spike–encoding lentiviruses were produced as previously described (Sampson et al., 2021). Briefly, HEK293T/17 cells at 60% confluency in 6-well plates were transfected with 250 ng p8.91 lentiviral packaging plasmid, 375 ng pCSFLW luciferase reporter, and 50 ng pcDNA3.1 expressing plasmid encoding either the WT or omicron spike gene. After 72 h, the supernatant was collected and filtered using 0.45 μm Millipore syringe filters and titrated on ACE2/TMPRSS2-transfected target cells. For neutralization assays, HEK293T/17 cells at ∼70% confluence in T75 flasks were transfected to express ACE-2 and TMPRSS2 by the addition of 600 μl Opti-MEM containing 2 μg ACE-2 pCAGGS plasmid, 150 ng TMPRSS2 pCAGGS plasmid, and 6.45 μl FuGENE-HD (Promega). Serum samples diluted over a twofold dilution series from 1:100 to 1:12,800 in 50 μl DMEM (10% FCS, 1% penicillin/streptomycin, and 1% L-glutamine) were incubated at 37°C for 1 h on Thermo Fisher Scientific Nunc FluoroNunc/LumiNunc 96-Well Plates with 50 μl virus (2 × 10^7^ RLU/ml) (except for cell-only control). Following incubation, 50 μl of transfected target HEK293T/17 cells was added at a density of 3 × 10^5^ cells/ml in fresh complete DMEM to all wells and incubated for 48 h at 37°C, 5% CO_2_. To develop plates, plates were flicked and then tapped to remove media, and then, 30 ml Bright-Glo (Promega) diluted 1:1 in PBS was added to each well. After 5 min at RT, plate luminescence was measured on a CLARIOstar Plus (BMG LABTECH) reader. The IC50 values were interpolated off of control-normalized, nonlinear curve transformations for each sample, in similar method to Ferrara and Temperton (2018).

### Data and statistical analyses

All statistical analyses were performed on GraphPad Prism 9.0 and 10.0. Medians were used as representative values for each mouse group. For the comparison of magnitudes in response between regimens, unpaired parametric statistical tests were completed (when data was normally distributed) unless otherwise specified; a “+” symbol was added to the upper left side of graphs that were analyzed using a nonparametric test for data that did not follow a normal distribution. To assess whether data were normally distributed, a Shapiro–Wilk test was performed. For the comparison of two groups, unpaired parametric t tests or nonparametric Mann–Whitney U tests were performed. When three or more groups were compared against each other, a parametric one-way ANOVA test or nonparametric Kruskal–Wallis test was performed. When multiple comparisons were made, parametric Tukey’s or Šidák’s test was performed depending on whether all groups were compared with each other, or with a specific a control group, respectively. For non-normal data, Dunn’s test was used. Correlation between two given variable readouts was assessed using the Pearson correlations. P values were symbolized as asterisks (* = P < 0.05, ** = P < 0.01, *** = P < 0.001, **** = P ≤ 0.0001). If data were represented in log form, data were converted to log prior to statistical analysis. Flow data were analyzed on FlowJo software (10.9.0) using the gating strategies described in Figs. S2 and S5.

### Online supplemental material

Fig. S1 shows the comparison of the immunogenicity of Ad-WT^IM^+Ad-o^IN^ and Ad-o^IN^ regimens. Fig. S2 shows the gating strategy for the flow cytometry completed for the experiment featured in Figs. 1, 2, and 9. Fig. S3 shows vaccine titration data in Syrian golden hamsters to identify a partially protective dose. Fig. S4 shows data from an additional experiment where the immunogenicity of heterologous regimens Ad-α^IM^+Ad-ο^IM^ and Ad-ο^IM^+Ad-α^IM^ is compared. Fig. S5 shows data from an additional cell fate mapping experiment where the prime-boost interval was 28 days. Table S1 shows the omicron S1 spike peptide stimulation pool used for IFNγ ELISpot assay.

## Supplementary Material

Table S1 shows the peptide pool for IFNγ ELISpot.

## Data Availability

The data generated in this study, as well as any additional information or reagents, are available from the corresponding author upon reasonable request.
