# Supplementary material for: Heterologous mucosal vaccine boosting enhances mucosal and systemic immunity by distinct mechanisms
Source: J Exp Med. 2025 Oct 30;223(1):e20241529. doi: 10.1084/jem.20241529 (PMC12574661; doi:10.1084/jem.20241529)
Supplement: Table S1 — shows the peptide pool for IFNγ ELISpot. [file jem_20241529_tables1.docx]

Table S1. **Peptide pool for IFNγ ELISpot**

(A) Peptides used (left column) for the stimulation of lung cells and splenocytes. S1-spanning peptides were included that contained amino acid mutations. The pool does not contain peptides that are completely homologous between WT and omicron (BA.1) spike. Peptides are 15 amino acids long and overlap by 11 amino acids.

| Omicron BA.1-specific S1 spike peptides | WT equivalent S1 spike-peptides |
| --- | --- |
| DLFLPFFSNVTWFHV | DLFLPFFSNVTWFHA |
| PFFSNVTWFHVISGT | PFFSNVTWFHAIHVS |
| NVTWFHVISGTNGTK | NVTWFHAIHVSGTNG |
| FHVISGTNGTKRFDN | FHAIHVSGTNGTKRF |
| SGTNGTKRFDNPVLP | HVSGTNGTKRFDNPV |
| KVCEFQFCNDPFLDH | KVCEFQFCNDPFLGV |
| FQFCNDPFLDHKNNK | FQFCNDPFLGVYYHK |
| NDPFLDHKNNKSWME | NDPFLGVYYHKNNKS |
| LDHKNNKSWMESEFR | LGVYYHKNNKSWMES |
| NNKSWMESEFRVYSS | YHKNNKSWMESEFRV |
| FKIYSKHTPINIVEP | FKIYSKHTPINLVRD |
| SKHTPINIVEPERDL | SKHTPINLVRDLPQG |
| PINIVEPERDLPQGF | PINLVRDLPQGFSAL |
| VEPERDLPQGFSALE | VRDLPQGFSALEPLV |
| RDLPQGFSALEPLVD | PQGFSALEPLVDLPI |
| SIVRFPNITNLCPFD | SIVRFPNITNLCPFG |
| FPNITNLCPFDEVFN | FPNITNLCPFGEVFN |
| TNLCPFDEVFNATRF | TNLCPFGEVFNATRF |
| PFDEVFNATRFASVY | PFGEVFNATRFASVY |
| CVADYSVLYNLAPFF | CVADYSVLYNSASFS |
| YSVLYNLAPFFTFKC | YSVLYNSASFSTFKC |
| YNLAPFFTFKCYGVS | YNSASFSTFKCYGVS |
| PFFTFKCYGVSPTKL | SFSTFKCYGVSPTKL |
| DEVRQIAPGQTGNIA | DEVRQIAPGQTGKIA |
| QIAPGQTGNIADYNY | QIAPGQTGKIADYNY |
| GQTGNIADYNYKLPD | GQTGKIADYNYKLPD |
| NIADYNYKLPDDFTG | KIADYNYKLPDDFTG |
| FTGCVIAWNSNKLDS | FTGCVIAWNSNNLDS |
| VIAWNSNKLDSKVSG | VIAWNSNNLDSKVGG |
| NSNKLDSKVSGNYNY | NSNNLDSKVGGNYNY |
| LDSKVSGNYNYLYRL | LDSKVGGNYNYLYRL |
| VSGNYNYLYRLFRKS | VGGNYNYLYRLFRKS |
| ERDISTEIYQAGNKP | ERDISTEIYQAGSTP |
| STEIYQAGNKPCNGV | STEIYQAGSTPCNGV |
| YQAGNKPCNGVAGFN | YQAGSTPCNGVEGFN |
| NKPCNGVAGFNCYFP | STPCNGVEGFNCYFP |
| NGVAGFNCYFPLRSY | NGVEGFNCYFPLQSY |
| GFNCYFPLRSYSFRP | GFNCYFPLQSYGFQP |
| YFPLRSYSFRPTYGV | YFPLQSYGFQPTNGV |
| RSYSFRPTYGVGHQP | QSYGFQPTNGVGYQP |
| FRPTYGVGHQPYRVV | FQPTNGVGYQPYRVV |
| YGVGHQPYRVVVLSF | NGVGYQPYRVVVLSF |
| HQPYRVVVLSFELLH | YQPYRVVVLSFELLH |
| LVKNKCVNFNFNGLK | LVKNKCVNFNFNGLT |
| KCVNFNFNGLKGTGV | KCVNFNFNGLTGTGV |
| FNFNGLKGTGVLTES | FNFNGLTGTGVLTES |
| GLKGTGVLTESNKKF | GLTGTGVLTESNKKF |
| NVFQTRAGCLIGAEY | NVFQTRAGCLIGAEH |
| TRAGCLIGAEYVNNS | TRAGCLIGAEHVNNS |
| CLIGAEYVNNSYECD | CLIGAEHVNNSYECD |
| AEYVNNSYECDIPIG | AEHVNNSYECDIPIG |
| PIGAGICASYQTQTK | PIGAGICASYQTQTN |
| GICASYQTQTKSHRR | GICASYQTQTNSPRR |
| SYQTQTKSHRRARSV | SYQTQTNSPRRARSV |
| QTKSHRRARSVASQS | QTNSPRRARSVASQS |
| HRRARSVASQSIIAY | PRRARSVASQSIIAY |
